# Screening of tarragon accessions based on physiological and phytochemical responses under water deficit

**DOI:** 10.1038/s41598-021-97388-z

**Published:** 2021-09-08

**Authors:** Hasan Mumivand, Amin Ebrahimi, Alireza Shayganfar, Hamid Hassaneian Khoshro

**Affiliations:** 1 Department of Horticultural Science, Faculty of Agriculture, Lorestan University, PO Box 465, Khorramabad, Iran; 2 Departments of Agriculture and Plant Breeding, Faculty of Agriculture, Shahrood University of Technology, Semnan, Iran; 3 Department of Horticultural Science and Landscape Engineering, Faculty of Agriculture, Malayer University, Malayer, Iran; 4 Dryland Agricultural Research Institute (DARI), Agriculture Research, Education and Extension Organization (AREEO), Maragheh, Iran

**Keywords:** Biochemistry, Plant sciences

## Abstract

In this study, screening of *Artemisia dracunculus* accessions was investigated under water deficit based on physiological and phytochemical traits. The results clearly indicated that water deficit significantly reduced the relative water content, chlorophyll, and carotenoid contents and increased malondialdehyde, electrolyte leakage, and antioxidant activities. The responses of tarragon accessions to water deficit, however, were inconsistent. The HPLC analysis revealed the presence of chlorogenic, syringic, ferulic, vanillic, chicoric, and *p*-coumaric acids as major phenolic acids, while quercetin and herniarin were detected as the predominant flavonoid and coumarin compounds in the extracts. Our findings revealed that the water deficit not only increased the amounts of herniarin, luteolin, apigenin, caffeic acid, and syringic acid, but also introduced quercetin that was not present under normal conditions in Estahbanat. Nevertheless, these results were highly impacted by the accession type. The results indicated that Hamadan, Varamin and Estahbanat accessions could be introduced as tolerant accessions. Given the very different responses of tarragon accessions to water deficit and the diversity between these accessions, the findings of the present study could be an effective step in identifying and achieving homogeneous, drought-tolerant and high-yield potential accessions, and may help tarragon breeding programs as well as development of cultivation.

## Introduction

A severe decrease in water availability most likely leads to drought stress as well as some unsatisfactory physiological and phytochemical changes in plants. Drought is the most common environmental stress and is considered as an important factor limiting crop production worldwide which reduces the production up to approximately 30–40% of the global agricultural fields^1^.

The herbaceous and perennial *Artemisia dracunculus,* which belongs to the Asteraceae family, the subfamily of *Radia*, and *Artemisia* genus, has woody and yellowish-green or brownish-green stalks of 30–150 cm in height, depending on the climate of its growth environment. It is one of the 20 most commonly grown herbs in Europe and is consumed as fresh, dry, and frozen products^2^. The main classes of secondary bioactive metabolites of tarragon include coumarins, flavonoids, and phenolic acids^3^. Previous studies have mostly focused on identifying essential oil compounds of tarragon and their diversity^4,5^. Some studies, however, have investigated the tarragon's polyacetylene derivatives, coumarins, flavonoids and, to a lesser extent, the sesquiterpene, vitamins, tannins, and alkaloids found in its aerial parts.

Identifying and screening plant germplasm tolerant to water deficit conditions would be the underlying objectives of relevant scientific research. Wild germplasms and native genotypes are valuable genetic resources for crucial physiological traits such as drought tolerance which could be identified and applied in breeding programs^6^. Obviously, among the different populations of a plant species, those that are more tolerant to water stress are better choices for arid and semi-arid areas^7^. The impact of water scarcity on plant yields and adverse changes of active substances of medicinal plants must be thoroughly evaluated. Any attempt to genetically modify drought tolerance using the existing genetic diversity largely requires an efficient screening or other functional approaches to be rapid and capable of evaluating plants at sensitive growth stages^8^.

Populations of individual species of medicinal plants that grow in different ecological conditions constitute inconsistent types in terms of quantity and quality of their active ingredients, which, in turn, leads to dissimilarities in the range of their medicinal as well as biological activities. Genetic flexibility of plant populations allows the emergence of diversity and gradually forms populations of the same species across dissimilar geographic regions, which largely differ in terms of developmental, physiological, chemical, and botanical activities^9^. For the reasons mentioned, native populations of medicinal plants are heterogeneous based on morpho-phenological and chemical properties^10^. Thus, if a medicinal plant is used in industry, homogeneous cultivars with an optimal level of active ingredient should be applied to meet World health organization (WHO) objectives for optimum cultivation of medicinal plants and production of safe, stable, and efficient raw materials^11^.

Although native tarragon accessions of Iran have already been collected and some of their qualitative and quantitative characteristics have been studied^12,13^, their tolerance to environmental stresses, especially drought stress, has not been assessed in detail. Obviously, some of these accessions, apart from their higher potential for cultivation, might be used in breeding programs as sources of stress resistance. This may require examining the relationship between drought tolerance and their physiological, morphological, and phytochemical markers. Accordingly, the present study could be a starting point for extensive scientific research on tarragon accessions to screen and introduce drought-tolerant cultivars in the future.

Given that Iran has a high rank in the world in terms of cultivation and production of this crop, considerable variation has been observed in the tarragon accessions in the country^12,13^. The core objectives of this study were screening twelve accessions of Iranian tarragon based on important physiological and biochemical traits relating to water-deficit tolerance followed by evaluating their secondary metabolite changes under water deficit.

## Results

### Variance analysis of physiological traits

The results of variance analysis of physiological traits revealed that water deficit stress had a significant effect on the measured traits (Supplementary Table 1). Significant differences were observed between different accessions of tarragon for all studied traits as well (Supplementary Table 2). In addition, the interaction effects of water deficit * accessions were significant for all studied traits except relative water content (Supplementary Table 3).

### Comparison of the mean physiological traits

**Figure 1:**
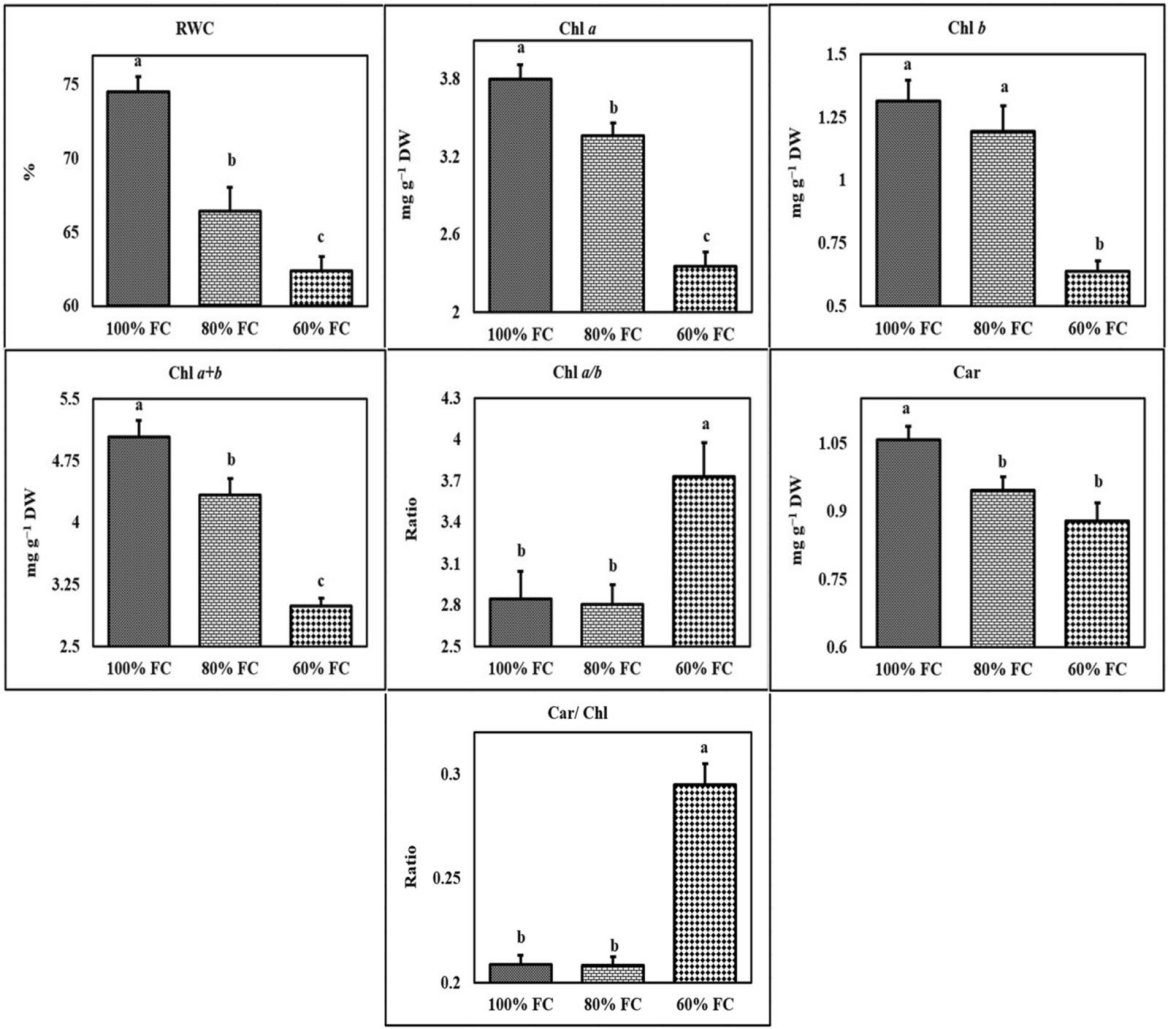
The effect of different levels of water deficit on physiological traits. The mean comparison was performed by LSD method at 5% probability. Columns with similar letters did not differ significantly. RWC: Relative water content; Chl *a*: Chlorophyll *a*; Chl *b*: Chlorophyll *b;* Chl *a* + *b*: Total chlorophyll; Chl*a*/Chl*b*: Chlorophyll *a/ b* ratio; Car: Carotenoid; Car/Chl: Carotenoid/total chlorophyll ratio.

**Figure 2:**
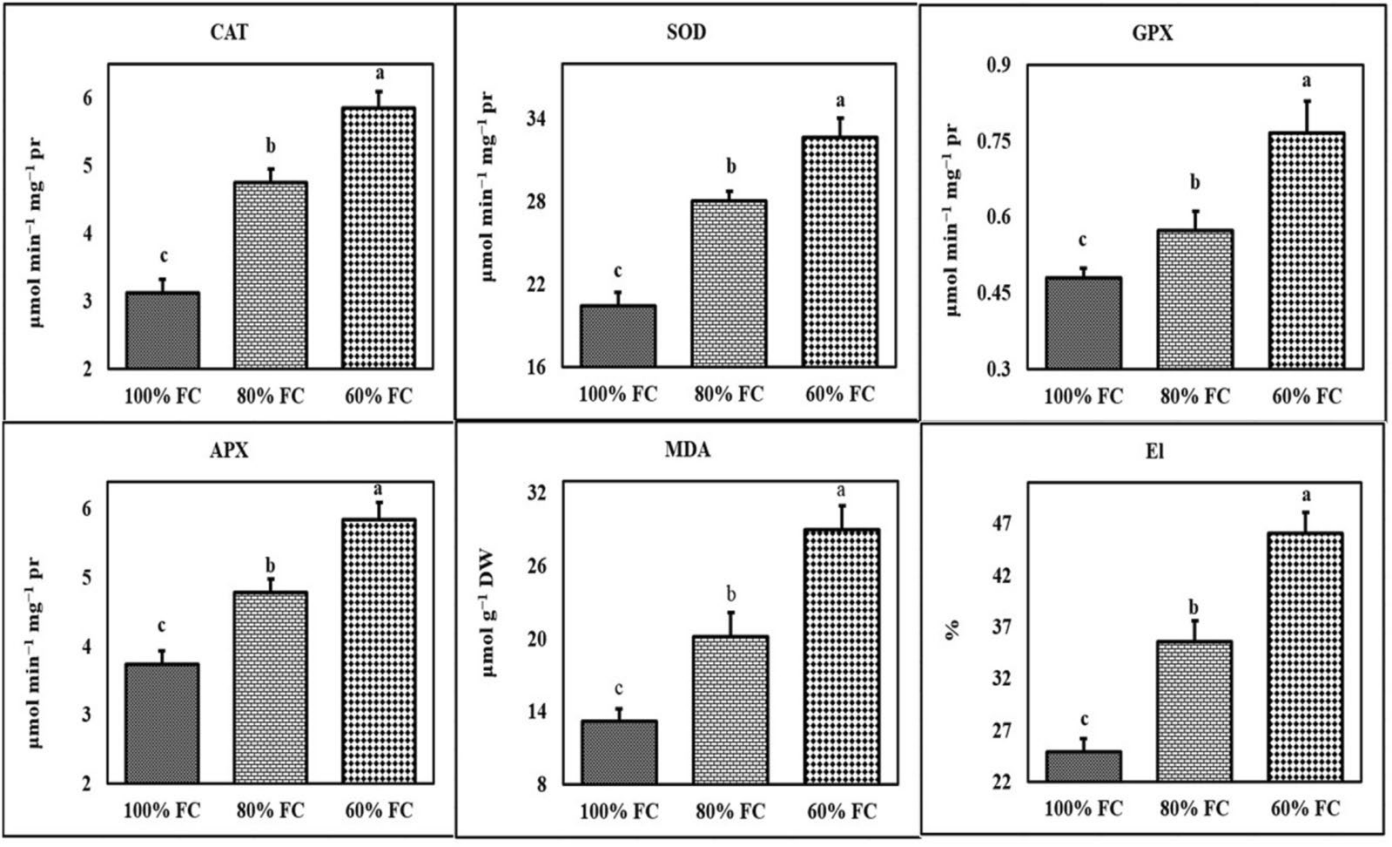
The effect of different levels of water deficit on physiological traits. The mean comparison was performed by LSD method at 5% probability. Columns with similar letters did not differ significantly. CAT: Catalase; SOD: Superoxide dismutase; GPX: Guaiacol peroxidase; APX: Ascorbate peroxidase; MDA: Malondialdehyde; El: Electrolyte leakage.

The relative water content of leaf under water deficit was significantly reduced whereby the highest relative water content (74.55%) was obtained under control condition and the lowest one (62.33%) was recorded under severe stress (Figs. 1, 2). There was also a significant difference in terms of relative water content between different tarragon accessions (Supplementary Table 1). Varamin, Hamadan, and Yazd accessions had the highest relative water content, while the lowest relative water content belonged to Isfahan, Abadeh, and Semirom (Supplementary Table 2). Examining the interaction between water deficit * accessions revealed that Hamadan accession had the highest content of chlorophyll *a*, chlorophyll *b*, and total chlorophyll under normal and mild stress conditions. On the other hand, Isfahan, Abadeh, and Semirom accessions had the lowest amount of these traits under severe stress conditions. Hamadan accession showed the highest content of carotenoid, while the lowest carotenoid content was recorded for Abadeh, Semirom, and Zarand accessions under drought stress. In general, a decline in chlorophyll *a, b*, and total chlorophyll content was observed under water deficit in most tarragon accessions, though the rate of reduction varied greatly in relation to the drought severity (Supplementary Table 3). Most of the accessions studied did not show a significant reduction in chlorophyll content under mild stress. In addition, concerning Yazd accession, there was no decrease in chlorophyll content even in severe stress treatments (Supplementary Table 3).

Despite falling carotenoid content under water deficit in most of the tarragon accessions, this valuable substance increased in Yazd, Varamin, and Hamadan accessions under water deficit. Carotenoid/chlorophyll ratio significantly rose in all tarragon accessions under severe drought stress, while most of the accessions (except Zarand) did not increase under mild stress (Supplementary Table 2). Examining the interaction effects of water deficit * accessions demonstrated that Varamin and Yazd accessions had the highest activity of superoxide dismutase (52.83 and 51.95 μmol min^−1^ mg^−1^ protein, respectively), catalase (9.24 and 9.23 μmol min^−1^ mg^−1^ protein, respectively), guaiacol peroxidase (1.44 and 1.29 μmol min^−1^ mg^−1^ protein, respectively), and ascorbate peroxidase (9.03 and 9.95 μmol min^−1^ mg^−1^ protein, respectively) under severe stress. The ascorbate peroxidase activity of Neishabour was also considerable under severe stress (7.74 μmol min^−1^ mg^−1^ protein). The lowest activity of superoxide dismutase was observed in Semirom, Abadeh, Kermanshah, and Birjand accessions under normal conditions. Semirom, Isfahan, Kermanshah, and Birjand also indicated the lowest catalase activity under control conditions. Semirom, Isfahan, and Zarand accessions had the lowest activity of ascorbate peroxidase under normal conditions. The trend of changes in antioxidant enzymes activity in all tarragon accessions under water deficit was incremental. On the other hand, the degree of activity changes in various accessions was somewhat different and in general, the activity of all enzymes was higher in Yazd, Varamin, and Hamadan concerning other studied accessions (Supplementary Tables 2 and 3).

The malondialdehyde content and electrolyte leakage percentage of the studied accessions also increased under water deficit. Among the investigated accessions, the highest values of these traits were found for Semirom, Abadeh and Isfahan. In general, the highest and lowest contents of malondialdehyde (44.40 and 10.35 nmol g^−1^ dry weight, respectively) were obtained in Semirom under severe stress and Isfahan under control conditions. The maximum and minimum percentage of electrolyte leakage (58.45% and 20.23%, respectively) under severe stress and control conditions was recorded for Abadeh accession (Supplementary Tables 2 and 3).

### Phytochemical traits

#### Antioxidant capacity

The results revealed that water deficit had a significant influence on antioxidant activities calculated through DPPH and FRAP tests (Supplementary Table 4). Various tarragon accessions also showed significant differences in terms of antioxidant capacity in both tests (Supplementary Table 5). However, in none of the applied methods, the interaction effect of water deficit * accessions on the antioxidant capacity of the extract was significant (Supplementary Table 6). Based on the mean comparison results, it was observed that in DPPH assay, IC50 methanol extract of tarragon declined under water deficit.

**Figure 3:**
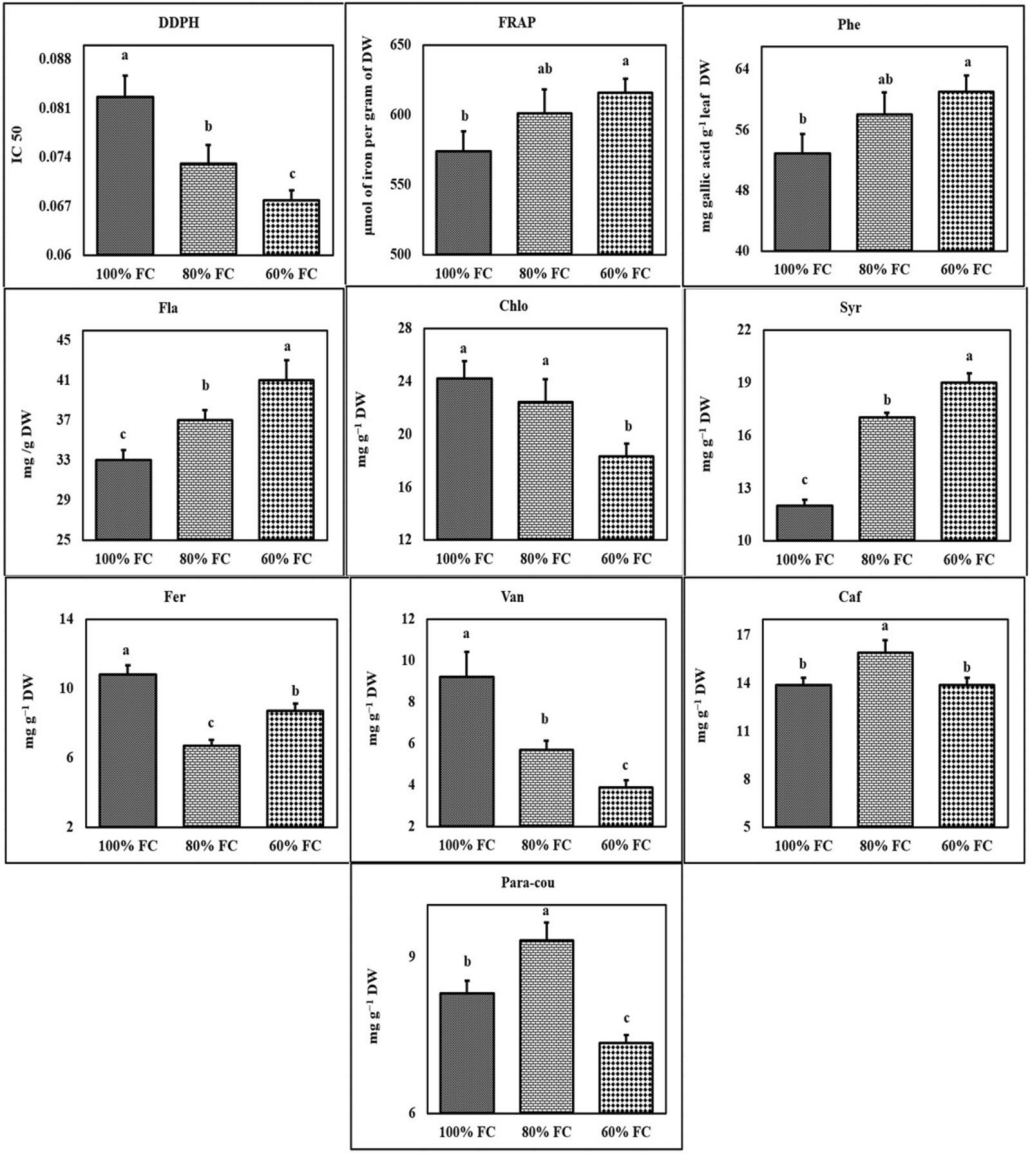
The effect of different levels of water deficit on phytochemical traits. The mean comparison was performed by LSD method at 5% probability. Columns with similar letters did not differ significantly. Phe: Total phenol; Fla: Total flavonoid; Chlo: Chlorogenic acid; Syr: Syringic acid; Fer: Ferulic acid; Van: Vanillic acid; Caf: Caffeic acid; *p*-cou: *p*-coumaric acid.

The highest and the lowest antioxidant capacity were observed under severe stress (IC50 = 0.076) and control treatment (IC50 = 0.082), respectively (Fig. 3). The highest and the lowest antioxidant capacity (616.16 μmol of iron per gram of dry weight and 574.9 μmol of iron per gram of dry weight, respectively) were also obtained under severe stress and control conditions (Fig. 3). In DPPH test, Birjand accession showed the highest antioxidant capacity (IC50 = 0.050) followed by Hamedan (IC50 = 0.056). The lowest antioxidant capacity also belonged to Varamin accession (IC50 = 0.103) followed by Abadea (IC50 = 0.102) (S5). Based on FRAP test, Birjand, Estahbanat, and Hamedan accessions enjoyed the highest antioxidant capacity (709.20, 690.80 and 686.00 μmol iron per gram of dry weight, respectively), while the lowest (402.30 μmol iron per gram of dry weight) belonged to Varamin accession (Supplementary Table 6).

#### Total phenol and flavonoid contents

The results of analysis of variance revealed that total phenolic and flavonoid contents of tarragon extract were significantly affected by water deficit (Supplementary Table 4). Significant differences were observed among all tarragon accessions in terms of total phenolic and flavonoids (Supplementary Table 5). The interaction effects of water deficit * accessions were also significant considering total phenol and flavonoid contents of the extract (Supplementary Table 6). The total phenol content of different tarragon accessions was influenced by water deficit in contrasting ways. Although there was an increase in total phenolic under water deficit in most accessions, which was significant only in Abadeh and Yazd accessions. The interaction effects of water deficit * accessions on the total flavonoids were also very similar to the total phenolic. The amount of total flavonoid rose in the most accessions under water deficit, but the difference between various levels of stress was significant only for Abadeh and Yazd accessions (Supplementary Table 6).

Finally, the highest total phenolic content (239.90 mg gallic acid g^−1^ dry weight) was observed in Abadeh accession under severe stress. The highest total flavonoid (152.25 mg g^−1^ dry weight) was obtained in Birjand accession under mild stress. Abadeh accession showed the lowest total phenols and flavonoids (16.92 mg gallic acid g^−1^ dry weight and 63.45 mg g^−1^ dry weight, respectively) under normal conditions (Supplementary Table 6).

#### Analysis of phenolic compounds of the extract

**Figure 4:**
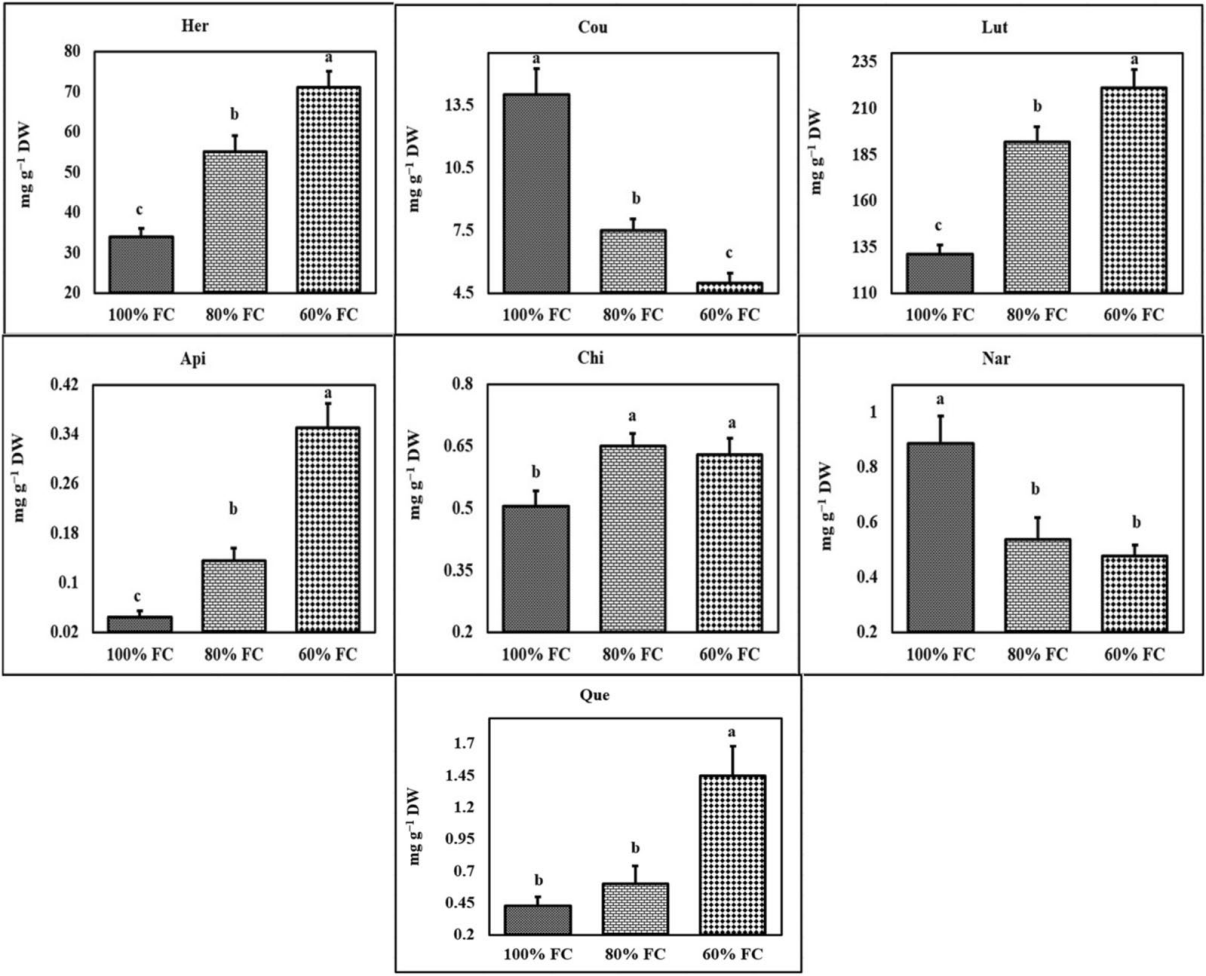
The effect of different levels of water deficit on phytochemical traits. The mean comparison was performed by LSD method at 5% probability. Columns with similar letters did not differ significantly. Her: Herniarin; Cou: Coumarin; Lut: Luteolin; Api: Apigenin; Chi: Chicoric acid; Nar: Naringenin; Que: Quercetin.

HPLC analysis of a methanolic extract of tarragon resulted in qualitative and quantitative identification of 14 phenolic compounds from three different groups. Chlorogenic acid, syringic acid, chicoric acid, caffeic acid, vanillic acid, ferulic acid, gallic acid, and *p*-coumaric acid were identified and measured as major plant phenolic acids. Qualitative and quantitative evaluation of important plant flavonoids including luteolin, quercetin, naringenin, and apigenin were performed as well. Coumarin and herniarin were identified and measured in tarragon accessions (Figs. 3, 4).

Variance analysis of phenolic compounds of tarragon extract indicated that drought stress had a significant influence on all these compounds (Supplementary Table 4). Significant differences were observed between the different accessions of tarragon for the detected phenolic compounds (Supplementary Table 5). The interaction effects of stress * accessions were also significant for all phenolic compounds (Supplementary Table 6).

Chlorogenic acid, syringic acid, and caffeic acid were the most important phenolic acids of Iranian tarragon accessions observed in all studied accessions. The highest amount of chlorogenic acid (38.48 mg g^−2^ dry weight) was obtained in Estabhanat accession while the lowest level of chlorogenic acid (4.90 mg g^−2^ dry weight) was found in Hamadan accession under normal conditions. The concentration of syringic acid was maximum (72.86 mg g^−2^ dry weight) in the Varamin, while the lowest syringic acid was (zero) recorded for Semirom accession (Supplementary Table 6). The highest and lowest amounts of caffeic acid (25.39 and 4.00 mg g^−2^ dry weight, respectively) belonged to Hamadan and Varamin accessions under normal conditions, respectively. Although the amount of vanillic acid and ferulic acid in the extracts of most of the accessions was considerable, these compounds were not found in some accessions. The highest amounts of ferulic acid and vanillic acid (242.20 and 26.71 mg g^−2^ dry weight, respectively) were obtained in Varamin and Estabhanat accessions under normal conditions, respectively. Chicoric acid was also observed only in Zarand, Birjand, and Kermanshah accessions in low amounts (Supplementary Table 6). Luteolin was the most important flavonoid compound of tarragon accessions extract, ranging from 40.11 mg g^−2^ dry weight in Zarand under normal conditions to 658.60 mg g^−2^ dry weight in Kermanshah under severe stress. The highest content of herniarin (124.60 mg g^−2^ dry weight) was recorded for Varamin under severe stress while the lowest (17.82 mg g^−2^ dry weight) belonged to Hamadan accession under normal conditions (Supplementary Table 6).

Coumarin was identified in all of the studied tarragon extracts except for Isfahan accession. The highest amount of this compound (46.63 mg g^−2^ dry weight) was obtained in Neyshabur under normal conditions (Supplementary Table 6). Naringenin was observed only in Neyshabour and Estahbanat accessions, with quercetin found only in Isfahan, Neyshabour, Birjand, and Hamedan accessions. Finally, apigenin was detected just in Semirom and Zarand accessions.

#### Correlation between phytochemical traits

The correlation results demonstrated that there was a significant correlation between the two methods of measuring antioxidant capacity (Supplementary Table 7). The total phenol was correlated with the total flavonoid (*r* = 0.95**). Also, these two traits were highly correlated with the amount of antioxidant capacity calculated using both methods. The antioxidant capacity of extract (FRAP) was positively correlated with the total phenol (*r* = 0.64**), total flavonoid (*r* = 0.70**), chlorogenic acid (*r* = 0.86**), and caffeic acid (*r* = 0.75**). However, there was a negative correlation between the antioxidant activity measured using FRAP method and syringic acid (*r* = − 0.62**), ferulic acid (*r* = − 0.71**), *p*-coumaric acid (*r* = − 0.60**), herniarin (*r* = − 0.68**), and luteolin (*r* = − 0.83**). The antioxidant capacity of the extract by DPPH method was not correlated with any of the polyphenolic compounds of the extract. There was a positive correlation between the total phenol and chlorogenic acid (*r* = 0.68**) and caffeic acid (*r* = 0.74**). Caffeic acid (*r* = 0.71**), and chlorogenic acid (*r* = 0.62**) were also correlated with total flavonoid (Supplementary Table 7).

## Discussion

According to the results of this study, various tarragon accessions had significant differences in relative water content of leaves (Supplementary Tables 1 and 2). Comparison of relative water content indicated that the accessions of Varamin, Hamadan, and Yazd with the highest relative water content were probably more drought-tolerant than other accessions.

The results of tarragon leaf pigment measurements showed that under severe drought stress, chlorophyll *a*, *b*, and total chlorophyll content diminished in all accessions (Supplementary Tables 2 and 3). The loss of leaf water not only impedes chlorophyll synthesis but also appears to cause chlorophyll degradation. Drought causes the chloroplast to break down and reduce the concentration of chlorophyll. Since both chlorophyll and proline are synthesized from a common precursor called glutamate, it could be stated that increased proline synthesis under drought stress leads to a reduction in chlorophyll synthesis^14^. The findings of this study suggest that different tarragon accessions somewhat vary in this respect and may exhibit different degrees of drought tolerance. In this study, the chlorophyll content at mild stress did not change compared to the control treatment, and severe stress lowered the content of this pigment (Figs. 1, 2). Reductions in chlorophyll *a, b*, and total chlorophyll content have been reported in several studies^15,16^. Miao et al. found that chlorophyll *a, b*, and total chlorophyll contents fell under extreme stress conditions, while the chlorophyll/carotenoid and chlorophyll *a*/*b* ratios rose^17^. Our findings demonstrated that carotenoid content of tarragon accessions had different responses to drought stress, which may qualify this trait as the main criterion for distinguishing the resistant accessions (Supplementary Tables 2 and 3).

In the present study, the activity of antioxidant enzymes activities increased in all accessions under drought stress, albeit with varying intensity. It seems that the high intensity of antioxidant enzymes activity in Varamin, Hamadan, and Yazd accessions reflect a high tolerance to drought stress. Hosseinpour et al. reported that the activity of superoxide dismutase, ascorbate peroxidase, polyphenol oxidase catalase, guaiacol peroxidase, and total protein and proline contents rose as a defense mechanism in *Echinacea purpurea* under drought stress conditions^18^.

A significant increase in peroxidation of cell membranes in leaves under drought stress could be due to increased production of oxygen free radicals under stress conditions causing their reaction with fatty acids of cell membranes and their oxidation^19^. The findings of the present study revealed that the content of malondialdehyde and electrolyte leakage (%) increased in all studied accessions under drought stress (Supplementary Table 3). However, in relation to the other accessions, Semirom, Isfahan, and Abadeh contained higher contents of malondialdehyde and electrolyte leakage, which may indicate their higher susceptibility to this abiotic stress. Numerous reports have shown that lipid peroxidation is more prevalent in drought-sensitive species and genotypes than in resistant stress^20^. Mohasseli et al. found that the chlorophyll and relative water content diminished steadily under drought stress. Malondialdehyde and electrolyte leakage (%) as well as H_2_O_2_ content significantly increased in *Melissa officinalis* as well^21^. Their results indicated that drought stress enhanced proline content and antioxidant enzymes, including catalase, ascorbate peroxidase, and polyphenol oxidase activities.

Identification of phenolic components of methanol extract via high-performance liquid chromatography indicated the presence of chlorogenic, syringic, chicoric, caffeic, vanillic, ferulic, and *p*-coumaric acids, as well as luteolin, quercetin, naringenin, apigenin, herniarin, and coumarin (Figs. 3, 4). Numerous studies have been carried out on the quantitative and qualitative identification of phenolic compounds of tarragon aerial parts^3,22–24^.

In the current study, the results of the free radical scavenging activity of tarragon extract using the methods of DPPH and FRAP revealed the high antioxidant capacity of the plant extracts (Figs. 3, 4). In addition, two different antioxidant capacity assays showed similar results, indicating some differences in the antioxidant capacity of various accessions (Figs. 3, 4). The significant correlation between total phenol content as well as total flavonoid content and the antioxidant capacity of the extract in both methods implies the important role of polyphenolic compounds in the antioxidant capacity of the extract (Supplementary Table 7). Positive correlations between polyphenols and antioxidant capacity of the extract have also been reported in several studies^25,26^. The results of Bettaieb et al. showed the scavenging activity IC50 and reducing power EC50 increased at moderate and decreased at severe stress. Liu et al. found that moderate and severe drought stress substantially reduced root and shoot dry weight, while the root/shoot ratio rose. In addition, with increasing drought, the percentage of salvianolic acid B, dihydrotanshinone I, cryptotanshinone, tanshinone I, and tanshinone IIA grew significantly and the percentage of rosmarinic acid decreased^27^. Their findings implied that the moderate level of drought may act as a suitable stimulus to enhance the amount of secondary plant metabolites.

In order to improve the quantity of active compounds and to homogenize them, superior plant varieties with the same genetic background should be developed^28^. The results of this study revealed that proper application of active compounds in the extract should be regarded as an important part of the breeding purposes. Among the tarragon accessions, there was a proper diversity along with high biological activity. Nowadays one of the most chief concerns regarding the cultivation of medicinal plants is the variations in quality and quantity of these plants under various environmental conditions. In the meantime, water deficit is one of the major problems in crop production in arid and semi-arid regions, including Iran^9,29^. Ghahremani et al. observed that the total phenolic content in leaf and flower, phenolic acid, flavonol, and quercetin in the flower of *Verbascum songaricum* increased as a defense mechanism under drought stress conditions^30^.

Drought stress is generally considered a limiting factor in agriculture and one of the main causes of crop yield decline. However, medicinal plants cultivated in semi-arid Mediterranean regions usually produce more biologically active compounds than similar plants in moist regions. The distribution of essential oil-rich aromatic plants is wider in arid regions compared to other climatic areas. Thus, secondary metabolites are likely to be effective in drought tolerance mechanisms by reducing transpiration. Although other factors might contribute to elevation of secondary compounds in semiarid regions, the main cause of increases in these metabolites is the differences in the amount of water available to the plant. Because the reactions that the plant exhibits in response to stress affect the entire metabolism to a large extent, the production and accumulation of secondary metabolites are also impacted^31,32^. Kleinwächter and Selmar revealed that water scarcity combined with high light intensity resulted in stomatal closure, thereby reducing carbon dioxide absorption and stabilization^31^.

In this study, total phenol, total flavonoid, and antioxidant capacity of methanolic extract of tarragon increased following drought stress (Figs. 3, 4). Given the effective role of polyphenols in protecting the plant against oxidative stress induced by drought, increasing the amount of these compounds in the plant under drought stress seems probable. Increased synthesis of secondary metabolites under drought stress has been reported in several studies^16,27,33^. There is ample evidence of growing levels of all secondary metabolites, comprising simple and complex phenols, terpenes, alkaloids, glycosides, etc., following drought stress. There is, arguably, no doubt about the increase in natural compounds under drought, but the plant growth and drug yield could be the primary cause of the decline in metabolic function^34,35^.

The findings of this study were in line with the results of other researchers. Rahimi et al. investigated the long-term influence of water deficit stress on the antioxidant capacity of *Mentha piperita* L. Their results suggested that water deficit stress reduced all morphological traits, while H_2_O_2_ and malondialdehyde content increased significantly under water deficit stress. It was also observed that water deficit stress had a significant effect on the content of polyphenol oxidase and superoxide dismutase. They found that the total phenol and flavonoid contents as well as the free antioxidant activity significantly diminished as well. They concluded that moderate stress would enhance the biochemical properties of peppermint and ultimately the plant resistance^36^. Similarly, in this study, the content of abscisic acid in the treated plants increased. On the other hand, increasing levels of total phenol and total flavonoids due to drought stress have been reported in many studies^20,36^.

Investigation of changes in the phenolic composition of extracts revealed that the amount of herniarin and luteolin rose in most tarragon accessions impacted by water deficit (Supplementary Tables 5 and 6). However, the magnitude of this increase in different accessions was affected by stress, such that the difference between various levels of stress in some accessions was not significant. For example, the increase of herniarin in Hamadan, Kermanshah, Semirom, Abadeh, and Anonymous was greater than that in other accessions. The ascending trend of the amount of luteolin in Kermanshah accession was more obvious as compared to the other studied accessions. In contrast, stress-induced changes in chlorogenic acid content in many tarragon accessions followed a falling pattern. The most severe reductions in chlorogenic acid were observed in Semirom, Abadeh, and Estahbanat accessions, while no significant changes were observed in Varamin, Yazd, Birjand, and anonymous accessions. The trend of caffeic acid changes under drought stress was upward in Neyshabour, Estahbanat, and Anonymous accessions, but in Hamedan, Kermanshah, Isfahan, Abadeh, and Zarand accessions, it followed a downward pattern (Supplementary Tables 5 and 6). Ritesh et al. found that the amount of artemisinin, artemisinic acid, dihydroartemisinic acid, and artemisia significantly dropped under drought stress^37^. The results of Bettaieb et al. indicated that the reactions of flavonoids content, cinnamic acids, and benzoic acids were inconsistent at different drought levels. The content of caffeic acid, vanillic acid, rosmarinic acid, *p*-coumaric acid, cinnamic acid, dihydroxybenzoic acid, ferulic acid, trans-cinnamic acid, quercetin 3-D-galactoside, quercetin, kaempferol, naringin, apigenin, amentoflavone, and flavone increased at moderate levels of drought stress. With the increase of stress intensity, however, the levels of these compounds diminished, but the content of chlorogenic acid, trans-2-Hydroxycinnamic acid and catechin increased. Meanwhile, the scavenging activity of IC50 and reducing the power of EC50 also grew at moderate intensity but fell at severe stress^33^.

Since Varamin and Hamadan accessions had almost the highest values in terms of most phytochemical traits, this part of the results confirmed the findings of the first and second parts of the experiment. Interestingly, Kermanshah, Hamadan, and Varamin accessions, which were selected as the most tolerant accessions according to the results of three different sections, did not have apigenin and naringenin. The question here is whether there is a relationship between the two compounds and drought tolerance or sensitivity. Mentioning this theory according to the results of this research is the only speculation that needs to be confirmed by detailed and coherent research. Meanwhile, in this study, the type and quantity of tarragon secondary metabolites were affected by water deficit.

The effect of drought stress is like a double-edged sword, because on the one hand it reduces plant dry matter while on the other hand, it increases the amount of secondary metabolites. Indeed, an increase in the amount of metabolites offsets the decline in the dry yield, so drought stress cannot be used as a suitable stimulus to enhance the secondary metabolites of tarragon, unless the tolerant cultivars are available^38,39^. Due to the tolerance of Hamadan, Varamin, and Kermanshah to drought stress, it seems that drought stress could be applied as a suitable stimulus to increase the amount of tarragon secondary metabolites or that of these accessions as suitable parents are used in the classic breeding programs and biotechnology approaches.

Interestingly, the measured traits in some genotypes did not change significantly under mild stress compared to normal conditions, making these cultivars suitable candidates for cultivation in areas that naturally suffer from mild water deficit stress. In addition, the quality and quantity of secondary metabolites were significantly impacted by water deficit stress, as the content of some of the measured compounds substantially increased under mild stress. The content of phenol, flavonoid, herniarin, luteolin, apigenin, gallic acid, and quercetin reached their highest values under severe stress conditions. Accordingly, when tarragon cultivation is done with the sole aim of producing secondary metabolites with different purposes, such as medicine and pharmacy, cosmetics, etc., some of these genotypes appear to be suitable candidates in regions with water shortages.

## Methods

### Tarragon accessions cultivation and applying treatments

The plant materials used in the present study consisted of 12 Iranian tarragon accessions. Considering their growth and yield, morphological and phytochemical characteristics and genetic diversity, these superior accessions were previously separated from 26 populations collected from the collection of Research Medicinal Plants Institute of Tehran Shahid Beheshti University^12,13^. The taxonomy of the studied plant was confirmed by a specialist botanist from the Research Medicinal Plants Institute of Tehran Shahid Beheshti University, Tehran, Iran. The plant materials were obtained under the supervision and permission of Shahid Beheshti University guidelines and according to institutional as well as national guidelines, with all authors complying with all local and national guidelines. Tarragon accessions used in our study were the most important accessions cultivated from the most common tarragon cultivation areas in Iran. The farm soil was analyzed before planting tarragon. The results of soil analysis and climatic conditions of tarragon cultivation area are presented in supplementary Tables 8 and 9, respectively. In April, the soil of the experimental plots was plowed and then, some organic matter was thoroughly mixed with the soils. The plant division method was applied foot propagating the plant. Providing a suitable medium for plant growth, the resultant propagules after the division of the plants were pruned and transferred to the farm on May 1.

In this study, the influence of water deficit was evaluated on 12 accessions of tarragon accessions in a split plot design via randomized complete block with three replications. Soil moisture treatments, comprising irrigation at 100 ± 5%, 80 ± 5% and 60 ± 5% of field capacity, as main-plots and tarragon accessions as sub-plots were considered. The main plots in each block (repetition) and in two different blocks (repetition) were separated by two and four-meter distances, respectively. The rows and the plants on each row were separated apart at 60 and 35 cm, respectively. Prior to drought stress treatments, all plants were well watered for good establishment. On June 6th, all plants were cut at 5 cm above the ground in order to achieve the plants with the same growth properties. The different drought treatments were initiated after two months of plant cultivation. A drip irrigation system was designed in the field and a water-meter was installed at the beginning of the water inlet path for measuring the volume of the water consumed. The control plants were continuously watered to the field capacity every 2–3 days during the whole cultivation period. The soil was maintained at 80 ± 5 and 60 ± 5% of the FC in moderate and severe water stressed treatments, respectively, by adding the desired amount of water to each treatment. The soil moisture content (v/v) up to a depth of 30 cm was measured daily by a Theta Probe device (manufactured by the German Company of Spectrum Technology Now) during both years of the experiment. When reaching the minimum allowable level, the amount of water shortage for each treatment was added to the plots. Furthermore, the conventional agricultural management (weed, pest and disease control) was provided during the entire period of plant growth and development.

### Sampling and physiological evaluation

The leaves of plants were harvested for evaluating physiological traits, with the samples kept in the freezer (− 80 °C) until measurement.

### Plants harvesting and extraction

The extract of the plant was provided using the maceration method^40^. For this purpose, 250 ml Erlenmeyer flasks containing 10 gr of milled dried plant, to which 100 ml of methanol–water solvent (80%) was added, were placed on the shaker for 72 h at room temperature. The contents of the Erlenmeyer flasks were then passed through a filter paper and the methanol solution was transferred to a rotary vacuum apparatus in order to remove the methanol from the extract. Then, the pure extracts were left in dark glass at 4 °C until analysis. The extract was analyzed for total phenolic content, total flavonoids, evaluation of non-enzymatic antioxidant activity, and HPLC analysis of phenolic compounds.

### Physiological characteristics

#### Relative water content

At the final stages of water stress, five leaves were selected from each plant and their fresh weight was determined. In order to identify the leaf weight in the turgor state, leaf fragments were exposed to low light intensity at 4 °C for 24 h in distilled water, aimed to absorb the leaf cells into the turgor state. Then, the swollen parts were carefully weighed again. Next, the leaves were dried at 75 °C for 24 h, their dry weights were measured, and the relative leaf water content (in percent) was obtained using the following formula^41^.

#### Chlorophyll and carotenoid contents

The content of different pigments, including chlorophyll *a*, *b*, total chlorophyll, and carotenoid contents were measured according to the method reported by Şükran et al.^42^. For this purpose, 0.125 g fresh leaf tissue with 10 ml of 80% acetone and 0.1 g calcium carbonate (to neutralize the acidic state of the intracellular fluid and prevent chlorophyll degradation) were crushed in a mortar. After centrifugation of the extract (16,000*g* for 10 min), the supernatant was applied to determine the pigment contents. Finally, the light absorbed at wavelengths of 663 nm (maximum chlorophyll *a* absorption), 645 nm (maximum light absorption of chlorophyll *b*), and 470 nm (maximum light absorption of carotenoids) was read using a UV–Vis spectrophotometer (UV-1800; Shimadzu Corporation, Kyoto, Japan).

#### Electrolyte leakage

Ten punched leaves were mixed with 10 ml distilled water. The containers were then shaken on the shaker for 24 h at 150 rpm whereby the electrolyte conductivity (EC0) was read. The solution containing the samples was then autoclaved at 120 °C for 20 min where electrolyte conductivity (EC1) was read again after cooling. Finally, the percentage of leaf electrolytes leakage (EL) was calculated by the following equation^43^:

$${\text{EL}}\, = \,\left( {{\text{EC}}0/{\text{EC1}}} \right)*{1}00.$$

#### Malondialdehyde content

Membrane lipid peroxidation was measured based on the concentration of malondialdehyde produced with damage to the membrane and its reaction with thiobarbituric acid, which forms a colored compound^44^. The absorbance of the mixture was measured by a UV–Vis spectrophotometer (UV-1800; Shimadzu Corporation, Kyoto, Japan) at two wavelengths of 532 nm and 600 nm. Note that the absorption at the second wavelength was the absorption of impure fats which should be less than the absorption at the first wavelength. In calculating the amount of malondialdehyde, the extinction coefficient (155 mM/cm) was also taken into account. The amount of malondialdehyde was expressed using the following equation:

$${\text{MDA}} = \left[ {\left( {{532}\,{\text{nm}}{-}{6}00\,{\text{nm}}} \right)/\left( {{\text{QD}}*{\text{QF}}} \right)} \right]*{\text{DF}}$$

QD = Cuvette diameter (1 cm), QF = Extinction coefficient (155 mmol/cm), DF = Dilution factor (20).

#### Antioxidant enzymes activity

An exact amount of 0.25 g of crushed tissue with digital scales was weighed and transferred to the falcons after which 2.5 ml of extraction buffer was added. All extraction procedures were performed at 4 °C on ice. After two minutes of vortex, the samples were centrifuged for 15 min at 4 °C with 28,000*g*. That extract could be used to measure the activity of catalase (CAT), ascorbate peroxidase (APX), guaiacol peroxidase (GPX), superoxide dismutase (SOD), and soluble proteins. Total protein was measured according to Bradford^45^. The activity of superoxide dismutase was measured spectrophotometrically and based on its inhibitory ability in the photochemical reduction of nitrobutetrazolium (NBT) at a wavelength of 560 nm^46^. Catalase activity (CAT) was measured at 25 °C using a spectrophotometer, which was set at 240 nm^48^. The activity of guaiacol peroxidase was measured at 470 nm as well. Ascorbate peroxidase activity was measured according to Ranieri et al.^48^. Note that the reaction between ascorbate peroxidase, ascorbic acid, and H_2_O_2_ produces dehydroascorbate, which can be read at 290 nm.

### Phytochemical evaluation

#### Total phenol content

The total phenol content was measured by Folin ciocalteu reagent^49^. First, 0.5 ml of the cyanoacetate reagent was mixed with 4 ml of 1 M Na2CO3 solution. After adding 0.5 ml of the solution to the plant extracts and mixing the compound thoroughly, the mixtures were incubated at room temperature for 15 min. Finally, the absorbance of the samples was measured by a Bio-Rad (Hercules, CA, USA) microtiter plate reader at 765 nm. All samples were analyzed in three replications with the total phenol content calculated based on the standard curve of gallic acid at concentrations of 0, 50, 100, 150, 250, and 500 mg l^−1^.

#### Total flavonoid content

The total flavonoid content was measured via aluminum chloride colorimetric method based on the guidelines set by Quettier -Deleu's (2000)^50^. For this purpose, 50 µl of the standard or solution, 400 µl of 2% aluminum chloride solution, and then 1200 µl of 5% potassium acetate solution were mixed. After 40 min incubation at 37 °C, the samples were read at 415 nm by a Bio-Rad (Hercules, CA, USA) microtiter plate reader. Three replications were applied for all samples and the standard flavonoid content of the samples was calculated through plotting the standard curve at concentrations of 0, 50, 100, 150, 250, and 500 mg l^−1^.

### Antioxidant capacity

#### Evaluation of antioxidant capacity by DPPH method

Antioxidant properties of methanol extract of tarragon accessions were evaluated by DPPH reagent (2,2-dipheny-l-picrylhydrazyl). In this method, the ability of the extract to trap DPPH radicals and transfer electron or radical hydrogen as well as to convert the DPPH radical form to the reduced DPPH-H form was evaluated. Determination of DPPH radical scavenging activity was performed according to the method of Choi et al.^51^ with the absorbance read at 517 nm using Bio-Rad (Hercules, CA, USA) microtiter plate reader.

#### Evaluation of antioxidant capacity by FRAP method

The FRAP method is based on the reduction of Fe^3+^-TPTZ (yellow) to Fe^2+^-TPTZ (blue) at low pH values. For this purpose, 180 µl of FRAP solution was added to 20 µl of methanol extract and kept at 37 °C for 8 min. The absorbance of the solutions at 593 nm was read by a Bio-Rad (Hercules, CA, USA) microtiter plate reader. The blank sample containing FRAP solution was also read. Also, Fe_2_SO_4_·7H_2_O solution was prepared at 0, 25, 50, 100, 150, 250, and 500 μg l^−1^ concentrations to draw the standard curve whose corresponding numbers were read^52^.

#### HPLC analysis

In this study, dried tarragon extract was dissolved in methanol and analyzed by HPLC Perkin Elmer series 200 Q/410 manufactured in the United States. The HPLC instrument had a quad-core 200-Q410 LCD pump, an auto-sampler, and a diode array UV spectrometer. The column utilized was phenyl 6-carbon reverse phase with a length of 25 cm, an inner diameter of 4.6 mm, and a particle diameter of 5 μm. The mobile phase consisted of 20 mM water and phosphoric acid, which entered the column in different proportions over 70 min (Table 1). Further, 10 µl of filtered methanol extracts were injected into the device. In order to identify the peak of each phenolic compound, the retention time in the sample was compared with the standard retention time of each injection. The type and amount of each material in the samples were determined based on the inhibition time and the area under the outlet curve, and further compared with the calibration curve obtained from different standard concentrations. The ultraviolet detector used was tuned at two wavelengths of 280 and 350 nm^53^.

**Table 1:** Mobile phase application injected into the HPLC device column.

Phase	Time (min)	Mobile phase velocity (ml min^−1^)	Water (%)	Phosphoric acid (%)
0	1	1	10	90
1	5	1	10	90
2	10	1	5	95
3	10	1	5	95
4	15	1	10	90
5	10	1	10	90

#### Statistical analysis of data

Variance analysis, mean comparison of treatments applying the least significant difference (LSD) at the 0.05 level of significance, and the correlation of phytochemical as well as physiological characteristics were performed using SPSS software 26 (IBM SPSS, Armonk, NY, USA^54^, https://www.ibm.com/products/spss-statistics). Excel 2013 was also applied to draw graphs.

## Supplementary Information

Supplementary Tables.

## Data Availability

The datasets used and/or analyzed during the current study are available from the corresponding author on reasonable request.
