# Supplementary material for: Screening of tarragon accessions based on physiological and phytochemical responses under water deficit
Source: Sci Rep. 2021 Sep 8;11:17839. doi: 10.1038/s41598-021-97388-z (PMC8426339; doi:10.1038/s41598-021-97388-z)
Supplement: Supplementary file 1 — Supplementary Tables. [file 41598_2021_97388_MOESM1_ESM.docx]

Table S1. Variance analysis of physiological traits of different tarragon accessions under different levels of water deficit (This experiment was performed as a factorial in the form of randomized complete blocks).

| SOV |  | df | RWC | Chl *a* | Chl *b* | Chl *a+b* | Chl*a*/Chl*b* | Car | Car/ Chl |
| --- | --- | --- | --- | --- | --- | --- | --- | --- | --- |
|  |  |  | Mean square |  |  |  |  |  |  |
| Block |  | 2 | 75.54 | 0.00024 | 0.0002 | 0.0031 | 0.126 | 0.0003 | 0.00013 |
| Factor A (Water deficit ) |  | 2 | 1348.46^**^ | 3.229^**^ | 1.118^*^ | 5.077^**^ | 9.701^**^ | 0.009^*^ | 0.0885^**^ |
| Error A |  | 4 | 11.205 | 0.00187 | 0.0015 | 0.0033 | 0.086 | 0.00075 | 0.00012 |
| Factor B (Accessions) |  | 11 | 690.728^**^ | 0.420^**^ | 0.030^**^ | 0.741^**^ | 0.086^*^ | 0.0455^**^ | 0.0016^**^ |
| AB |  | 22 | 35.506^ns^ | 0.033^**^ | 0.0055^**^ | 0.051** | 0.119^**^ | 0.0043^**^ | 0.0008 |
| Error B |  | 66 | 26.224 | 0.00240 | 0.00067 | 0.003 | 0.04 | 0.0007 | 0.00016 |

RWC: Relative water content; Chl *a*: Chlorophyll *a*; Chl *b*: Chlorophyll *b;* Chl *a+b*: Total chlorophyll; Chl*a*/Chl*b*: Chlorophyll *a/ b* ratio; Car: Carotenoid; Car/ Chl: Carotenoid/ Total chlorophyll ratio

* and ** Significantly at the probability level of %5 and %1, respectively.

Table S1 (continued).

| SOV |  | df | CAT | SOD | GPX | APX | MDA | El |
| --- | --- | --- | --- | --- | --- | --- | --- | --- |
|  |  |  | Mean square |  |  |  |  |  |
| Block |  | 2 | 0.062 | 0.987 | 0.015 | 0.02 | 3.867 | 25.613 |
| Factor A (Water deficit ) |  | 2 | 30.059^**^ | 41.331^**^ | 0.462^**^ | 19.524^**^ | 911.235^**^ | 4030.882^**^ |
| Error A |  | 4 | 0.5 | 2.297 | 0.0076 | 0.016 | 2.61 | 7.021 |
| Factor B (Accessions) |  | 11 | 3.159^**^ | 43.093^**^ | 0.075^**^ | 1.858^**^ | 22.894^**^ | 140.634^**^ |
| AB |  | 22 | 0.724^**^ | 16.268^**^ | 0.018^**^ | 0.478^**^ | 9.864^**^ | 48.146^**^ |
| Error B |  | 66 | 0.024 | 2.259 | 0.0055 | 0.089 | 1.747 | 19.539 |

CAT: Catalase; SOD: Superoxide dismutase; GPX: Guaiacol peroxidase; APX: Ascorbate peroxidase; MDA: Malondialdehyde; El: Electrolyte leakage.

* and ** Significantly at the probability level of %5 and %1, respectively.

Table S2. The mean comparison effects of water deficit on physiological traits for different tarragon accessions

| Physiological traits | | | | | | | | | | | | | |  |
| --- | --- | --- | --- | --- | --- | --- | --- | --- | --- | --- | --- | --- | --- | --- |
| Accessions | RWC | Chl *a* | Chl *b* | Chl *a+b* | Chl*a*/Chl*b* | Car | Car/ Chl | CAT | SOD | GPX | APX | MDA | EL | |
| Isfahan | 57.72 e | 2.94 h | 0.96 f | 3.9 gh | 3.2 abc | 0.93 e | 0.25 a | 4.08 gh | 24.84 ef | 0.45 e | 4.56 de | 22.11 b | 43.29 a | |
| Abadeh | 57.43 e | 2.91 h | 0.93 f | 3.84 f | 3.25 ab | 0.9 e | 0.25 ab | 3.9 h | 23.43 fg | 0.6 d | 4.8 cd | 26.46 a | 39.05 bc | |
| Semirom | 58.9 de | 2.91 h | 1.02 e | 3.96 g | 2.96 d | 0.9 e | 0.24 b | 3.9 h | 22.53 g | 0.6 d | 4.08 f | 26.76 a | 40.75 ab | |
| Neishabour | 70.48 b | 3.54 c | 1.14 bc | 4.71 c | 3.08 bcd | 1.02 cd | 0.22 c | 5.61 c | 31.56 c | 0.63 d | 6 b | 19.32 cd | 31.34 ef | |
| Zarand | 64.25 c | 3.36 de | 1.14 bc | 4.5 d | 3.14 a-d | 0.93 e | 0.21 c | 4.23 g | 25.17 e | 0.6 d | 4.65 de | 27.27 a | 36.33 cd | |
| Estahbanat | 70.27 b | 3.21 f | 1.08 d | 4.32 e | 3.08 bcd | 0.9 e | 0.21 c | 4.83 e | 27.71 d | 0.6 d | 5.13 c | 22.62 b | 35.05 cde | |
| Anonymous | 62.96 cd | 3.33 d | 1.11 cd | 4.5 d | 3.17 abc | 0.99 d | 0.22c | 5.34 d | 30.36 c | 0.69 d | 5.16 c | 21.03 bc | 34.27 de | |
| Birjand | 63.46 cd | 3.1 ef | 1.14 c | 4.44 d | 3.03 cd | 1.05 c | 0.24 ab | 4.56 f | 24.63 ef | 0.72 cd | 4.95 cd | 23.1 b | 35.47 cde | |
| Kermanshah | 66.49 bc | 3.09 g | 0.96 ef | 4.08 f | 3.29 a | 0.93 e | 0.24 b | 4.53 f | 24.09 ef | 0.42 e | 4.32 ef | 34.01 a | 36.33 cd | |
| Varamin | 82.08 a | 3.6 c | 1.2 b | 4.8 b | 3.02 cd | 1.4 b | 0.24 b | 6.78 a | 38.7 a | 0.99 a | 6.33 ab | 18.45 d | 30.03 f | |
| Yazd | 80.68 a | 3.69 b | 1.17 b | 4.89 b | 3.15 a-d | 1.14 b | 0.24 b | 6.84 a | 37.71 a | 0.81 bc | 6.51 a | 19.56 cd | 32.5 def | |
| Hamadan | 78.59 a | 4.2 a | 1.32 a | 5.55 a | 3.18 abc | 1.29 a | 0.24 b | 6.21 b | 35.16 b | 0.84 b | 5.97 b | 20.76 bcd | 32.08 ef | |

RWC: Relative water content; Chl *a*: Chlorophyll *a*; Chl *b*: Chlorophyll *b;* Chl *a+b*: Total chlorophyll; Chl*a*/Chl*b*: Chlorophyll *a/ b* ratio; Car: Carotenoid; Car/ Chl: Carotenoid/ Total chlorophyll ratio; CAT: Catalase; SOD: Superoxide dismutase; GPX: Guaiacol peroxidase; APX: Ascorbate peroxidase; MDA: Malondialdehyde; El: Electrolyte leakage.

*Mean comparison was performed by LSD method at 5% probability. Columns with similar letters did not differ significantly.

Table S3. The mean comparison of interaction effects of different water deficit levels and tarragon accessions on physiological traits.

| Physiological traits | | | | | | | | | | | | | | | | | | | | | | | | | | |  |  |  |  |
| --- | --- | --- | --- | --- | --- | --- | --- | --- | --- | --- | --- | --- | --- | --- | --- | --- | --- | --- | --- | --- | --- | --- | --- | --- | --- | --- | --- | --- | --- | --- |
| 100% of Field capacity | | | | | | | | | | | | | | | | | | | | | | | | | | |  |  |  |  |
| Accessions | RWC | Chl *a* | | Chl *b* | | Chl *a+b* | | Chl*a*/Chl*b* | | Car | | Car/ Chl | | CAT | | SOD | | GPX | | APX | | MDA | | | EL | | | |  |  |
| Isfahan | 63.61 j-n | 3.42 jk | | 1.23 ghi | | 4.68 lm | | 2.79 g-j | | 0.96 i-p | | 0.21 g-k | | 2.88 n | | 19.17 uvw | | 0.36 pq | | 3.33 mn | | 10.35 p | | | 31.2 k-p | | | |  |  |
| Abadeh | 60.17 m-q | 3.42 jk | | 1.14 hij | | 4.59 mn | | 2.96 fg | | 0.93 l-r | | 0.20 g-k | | 3.06n | | 18.27 vw | | 0.57 i-n | | 3.90 klm | | 15.87 lmn | | | 20.23 r | | | |  |  |
| Semirom | 72.25 e-i | 3.51 hij | | 1.23 ghi | | 4.77 jkl | | 2.79 g-j | | 1.02 f-j | | 0.21 ghi | | 2.79 n | | 17.61 w | | 0.42 nq | | 2.76 n | | 14.49 m-p | | | 25.14 o-r | | | |  |  |
| Neishabour | 78.92 b-e | 3.78 ef | | 1.23 ghi | | 5.67 fgh | | 3.01 efg | | 1.05 e-i | | 0.21 g-j | | 3.03 n | | 22.5 q-t | | 0.36 pq | | 4.44 jkl | | 11.91 nop | | | 21.86 qr | | | |  |  |
| Zarand | 70.97 e-j | 4.05 c | | 1.47 ab | | 5.52 c | | 2.74 g-j | | 0.99 h-m | | 0.18 l | | 3.18 mn | | 20.34 tuv | | 0.51 l-q | | 3.45 mn | | 18.03 klm | | | 22.3 qr | | | |  |  |
| Estahbanat | 75.73 c-g | 3.39 jkl | | 1.23 gh | | 4.62 lmn | | 2.74 g-j | | 0.93 n-s | | 0.20 i-l | | 3.15 mn | | 20.67 s-v | | 0.36 opq | | 3.96 klm | | 14.61 mno | | | 27.09 m-r | | | |  |  |
| Anonymous | 69.98 f-k | 3.60 ghi | | 1.29 efg | | 4.89 hij | | 2.80 g-j | | 1.02 g-l | | 0.20 g-k | | 3.18 mn | | 21.57 r-u | | 0.57 j-o | | 4.32 jkl | | 12.45 nop | | | 24.88 pqr | | | |  |  |
| Birjand | 65.77 i-m | 3.75 ef | | 1.38 cd | | 5.16 ef | | 2.71 g-j | | 1.14 cd | | 0.22 gh | | 3.03 n | | 19.02 vw | | 0.63 g-m | | 3.93 klm | | 12.93 nop | | | 25.79 n-r | | | |  |  |
| Kermanshah | 74.83 c-h | 3.60 ghi | | 1.23 gh | | 4.83 ijk | | 2.92 gh | | 1.02 g-l | | 0.21 g-j | | 2.91 n | | 18.42 vw | | 0.30 q | | 3.63 lm | | 13.65 nop | | | 25.59 n-r | | | |  |  |
| Varamin | 87.8 a | 3.81 def | | 1.29 efg | | 5.10 efg | | 2.92 ghi | | 1.11 def | | 0.21 ghi | | 3.51 m | | 23.40 pqr | | 0.60 h-n | | 3.87 klm | | 10.3 p | | | 25.58 n-r | | | |  |  |
| Yazd | 86.77 ab | 3.93 cd | | 1.38 bcd | | 5.31 d | | 2.80g-j | | 1.11 cde | | 0.21 g-j | | 3.54 m | | 22.95 qrs | | 0.42 n-q | | 3.72 klm | | 10.92 op | | | 23.89 qr | | | |  |  |
| Hamadan | 87.71 a | 4.56 a | | 1.50 a | | 6.09 a | | 3.00 efg | | 1.26 b | | 0.21 g-k | | 3.21 mn | | 21.81 rst | | 0.54 j-o | | 3.63 lm | | 13.62 nop | | | 25.58 n-r | | | |  |  |
| 80% of Field capacity | | | | | | | | | | | | | | | | | | | | | | | | | | |  |  |  |  |
| Isfahan | 55.17 opq | 3.27 lmn | | 1.08 jk | | 4.38 o | | 2.98 efg | | 0.93 l-s | | 0.21 ghi | | 4.44 jkl | | 26.73 l-o | | 0.45 m-q | | 4.86 hij | | 19.44 kl | | | 43.9 cde | | | |  |  |
| Abadeh | 59.41 m-q | 3.21 mn | | 1.14 j | | 4.35 o | | 2.83 g-j | | 0.90 o-s | | 0.21 g-j | | 4.14 l | | 24.69 n-q | | 0.63 g-m | | 4.86 hij | | 29.28 fgh | | | 38.49 e-i | | | |  |  |
| Semirom | 52.09 q | 3.18 n | | 1.23 gh | | 4.44 o | | 2.59 ij | | 0.90 p-s | | 0.20 h-k | | 4.29 kl | | 22.95 qrs | | 0.66 e-l | | 4.44 ijk | | 21.39 jk | | | 42.09 c-g | | | |  |  |
| Neishabour | 70.86 e-j | 3.90 de | | 1.32 def | | 5.22 de | | 2.95 fg | | 0.99 i-n | | 0.19 jkl | | 6.24 e | | 33.51 fg | | 0.66 f-l | | 6.00 ef | | 18.03 klm | | | 32.32 j-o | | | |  |  |
| Zarand | 66.81 h-m | 3.48 ijk | | 1.26 efg | | 4.77 jkl | | 2.72 g-j | | 0.96 j-q | | 0.20h-k | | 4.68 ijk | | 26.73 k-o | | 0.57 i-n | | 4.83 hij | | 25.89 gh | | | 38.17 e-k | | | |  |  |
| Estahbanat | 69.04 g-l | 3.51 hij | | 1.32 de | | 4.82 ijk | | 2.61 hij | | 0.90 p-s | | 0.19 kl | | 5.25 gh | | 31.2 hi | | 0.66 f-l | | 5.25 gh | | 21.63 ijk | | | 34.58 h-l | | | |  |  |
| Anonymous | 56.98 n-q | 3.63 gh | | 1.32 def | | 4.95 ghi | | 2.73 g-j | | 1.02 f-k | | 0.21 g-k | | 5.88 ef | | 31.38 gh | | 0.69 e-k | | 5.73 fg | | 20.31 k | | | 35.13 g-l | | | |  |  |
| Birjand | 63.42 j-o | 3.48 hij | | 1.35 de | | 4.86 ijk | | 2.58 j | | 1.08 d-g | | 0.22 g | | 5.22 gh | | 25.62 m-p | | 0.66 e-l | | 5.22 gh | | 20.7 k | | | 34.01 h-m | | | |  |  |
| Kermanshah | 65.76 i-m | 3.36 klm | | 1.14 ij | | 4.50 no | | 2.92 ghi | | 0.90 p-s | | 0.20 i-l | | 5.25 gh | | 24.42 opq | | 0.42 n-q | | 4.41 ijk | | 25.68 hi | | | 34.9 g-l | | | |  |  |
| Varamin | 80.76 a-d | 3.84 de | | 1.32 de | | 5.19 de | | 2.86 g-j | | 1.08 d-h | | 0.21 g-j | | 7.62 c | | 39.93 c | | 0.93 c | | 6.15 ef | | 19.5 kl | | | 27.94 l-q | | | |  |  |
| Yazd | 81.63 abc | 3.87 de | | 1.29 efg | | 5.19 de | | 2.98 efg | | 1.11 def | | 0.21 ghi | | 7.74 c | | 38.25 cd | | 0.72 d-j | | 6.96 c-d | | 19.38 kl | | | 32.58 j-n | | | |  |  |
| Hamadan | 75.02 c-h | 4.35 b | | 1.44 abc | | 5.82 b | | 3.00 efg | | 1.23 b | | 0.21 ghi | | 7.11 d | | 36.51 de | | 0.78 c-h | | 6.63 de | | 18.63 klm | | | 33.31 i-m | | | |  |  |
| 60% of Field capacity | | | | | | | | | | | | | | | | | | | | | | | | | | |  |  |  |  |
| Isfahan | 54.38 pq | | 2.07 s | | 0.54 p | | 2.61 v | | 3.84 abc | | 0.90 p-s | | 0.34 a | | 4.95 hi | | 28.11 h-k | | 0.51 k-p | | 5.46 fgh | | 36.57 bc | | | 54.78 ab |  | | |  |
| Abadeh | 52.66 q | 2.07 s | | 0.51 p | | 2.61 v | | 3.95 ab | | 0.57 st | | 0.33 ab | | 4.56 i-l | | 27.30 j-m | | 0.66 f-l | | 5.67 fg | | 34.29 cde | | 58.45 a | | | |  | | |
| Semirom | 52.37 q | 2.07 s | | 0.57 op | | 2.76 v | | 3.51 cd | | 0.51 t | | 0.30 cd | | 4.59 ijk | | 27.06 j-n | | 0.75 d-i | | 5.07 ghi | | 44.4 a | | 55.04 ab | | | |  | | |
| Neishabour | 61.65 k-p | 2.94 o | | 0.90 m | | 3.87 q | | 3.27 def | | 0.99 i-o | | 0.25 f | | 7.59 c | | 35.7 cd | | 0.90 cd | | 7.74 b | | 28.02 fgh | | 39.82 d-i | | | |  | | |
| Zarand | 54.98 pq | 2.58 q | | 0.66 no | | 3.24 t | | 3.95 ab | | 0.57 rst | | 0.27 ef | | 4.83 hij | | 28.44 i-l | | 0.72 d-j | | 5.67 fg | | 37.89 bc | | 48.55 bc | | | |  | | |
| Estahbanat | 66.03 i-m | 2.76 p | | 0.69 n | | 3.48 s | | 3.89 ab | | 0.57 q-t | | 0.25 f | | 6.12 e | | 34.8 ef | | 0.78 c-g | | 6.15 ef | | 31.65 def | | 43.5 c-f | | | |  | | |
| Anonymous | 61.91 k-p | 2.94 o | | 0.72 n | | 3.69 r | | 3.99 ab | | 0.96 k-r | | 0.25 f | | 6.96 d | | 38.19 cd | | 0.86 cde | | 5.43 fgh | | 30.33 ef | | 42.82 c-f | | | |  | | |
| Birjand | 61.18 l-p | 2.61 pq | | 0.69 n | | 3.33 t | | 3.79 abc | | 0.96 j-q | | 0.29 de | | 5.43 fg | | 29.46 hij | | 0.81 c-f | | 5.73 fg | | 32.7 bcd | | 46.64 cd | | | |  | | |
| Kermanshah | 58.87 m-q | 2.31 r | | 0.57 p | | 2.88 u | | 4.03 a | | 0.93 m-s | | 0.32 bc | | 5.49 fg | | 29.43 hij | | 0.57 i-n | | 4.92 hij | | 38.7 b | | 48.52 bc | | | |  | | |
| Varamin | 77.69 c-f | 3.15 n | | 0.96 lm | | 4.11 p | | 3.30 de | | 1.20 bc | | 0.29 d | | 9.24 a | | 52.83 a | | 0.144 a | | 9.03 a | | 25.5 hij | | 36.6 f-k | | | |  | | |
| Yazd | 73.64 c-i | 3.24 lmn | | 0.57 m | | 4.14 p | | 3.68 bc | | 1.12 b | | 0.30 d | | 9.24 a | | 51.96 a | | 0.129 ab | | 8.85a | | 28.38 fgh | | 41.02 d-h | | | |  | | |
| Hamadan | 73.03 d-i | 3.69 fg | | 1.02 kl | | 4.74 klm | | 3.55 cd | | 1.41 a | | 0.29 d | | 8.31 d | | 47.22 b | | 0.111 b | | 7.62 bc | | 30.09 efg | | 37.36 e-k | | | |  | | |

RWC: Relative water content; Chl *a*: Chlorophyll *a*; Chl *b*: Chlorophyll *b;* Chl *a+b*: Total chlorophyll; Chl*a*/Chl*b*: Chlorophyll *a/ b* ratio; Car: Carotenoid; Car/ Chl: Carotenoid/ Total chlorophyll ratio; CAT: Catalase; SOD: Superoxide dismutase; GPX: Guaiacol peroxidase; APX: Ascorbate peroxidase; MDA: Malondialdehyde; El: Electrolyte leakage. *Mean comparison was performed by LSD method at 5% probability. Columns with similar letters did not differ significantly.

| SOV | df | DPPH (IC50) | FRAP | Phe | Fla | Chlo | Fer | Caf | Van | Syr |
| --- | --- | --- | --- | --- | --- | --- | --- | --- | --- | --- |
|  |  |  |  | Mean square |  |  |  |  |  |  |
| Block | 2 | 0.000065 | 8567.456 | 53.999 | 6.389 | 27.216 | 0.131 | 2.608 | 0.32 | 0.106 |
| Factor A (Water deficit) | 2 | 0.002** | 15710.244* | 1589.694* | 878.679* | 330.358** | 130.402** | 35.462** | 254.971** | 443.543** |
| Error A | 4 | 0.000015 | 2152.786 | 192.208 | 93.033 | 11.544 | 0.034 | 1.312 | 0.022 | 0.126 |
| Factor B (Accessions) | 11 | 0.0026** | 78777.577** | 1512.48** | 793.022** | 431.124** | 4821.754** | 125.209** | 454.617** | 1955.175** |
| AB | 22 | 0.000012^ns^ | 2496.027^ns^ | 427.7** | 94.722** | 107.134** | 143.504** | 67.809** | 58.965** | 72.032** |
| Error B | 66 | 0.000036 | 1590.483 | 75.73 | 85.507 | 6.282 | 0.077 | 1.725 | 0.235 | 0.799 |

Table S4. Variance analysis of phytochemical traits of different tarragon accessions under different levels of water deficit (This experiment was performed as a factorial in the form of randomized complete blocks).

Phe: Total phenol; Fla: Total flavonoid; Chlo: Chlorogenic acid; Fer: Ferulic acid; Caf: Caffeic acid; Van: Vanillic acid; Syr: Syringic acid.

* and ** Significantly at the probability level of %5 and %1, respectively.

Table S4 (continued).

| SOV | df | *p*-cou | Her | Cou | Lut | Api | Chi | Gall | Nar | Que |
| --- | --- | --- | --- | --- | --- | --- | --- | --- | --- | --- |
|  |  |  |  | Mean square |  |  |  |  |  |  |
| Block | 2 | 0.094 | 4.052 | 0.306 | 89.798 | 0.056 | 0.032 | 0.192 | 0.07 | 0.10 |
| Factor A (Water deficit) | 2 | 44.94** | 12663.6** | 756.059** | 76072.7** | 0.82** | 0.25* | 0.11 | 1.73** | 9.10** |
| Error A | 4 | 0.203 | 6.598 | 0.249 | 11.371 | 0.029 | 0.11 | 0.09 | 0.08 | 0.24 |
| Factor B (Accessions) | 11 | 694.772** | 5326.232** | 799.66** | 268189.9** | 1.88** | 16.66*** | 40.58** | 19.89** | 18.72** |
| AB | 22 | 48.315** | 500.641** | 159.5** | 12180.04** | 0.46** | 520** | 0.197 | 3.04** | 1.85** |
| Error B | 66 | 0.306 | 4.249 | 0.198 | 51.722 | 0.037 | 11.55** | 0.166 | 0.05 | 0.17 |

*p*-cou: *p*-coumaric acid; Her: Herniarin; Cou: Coumarin; Lut: Luteolin; Api: Apigenin; Chi: Chicoric acid; Gall: Gallic acid; Nar: Naringenin; Que: Quercetin

* and ** Significantly at the probability level of %5 and %1, respectively.

Table S5. The mean comparison effects of water deficit on phytochemical traits for different tarragon accessions.

| Phytochemical traits | | | | | | | | | |
| --- | --- | --- | --- | --- | --- | --- | --- | --- | --- |
| Accessions | DPPH (IC50) | FRAP | Phe | Fla | Chlo | Fer | Caf | Van | Syr |
| Isfahan | 0.072 cd | 509.48 d | 168.30 bcd | 105.3 cd | 15.91 e | 17.75 b | 11.44 f | 9.89 d | 13.58 ef |
| Abadeh | 0.102 a | 617.85 bc | 167.94 bcd | 103.8 cd | 26.15 b | 0.14 gh | 16.58 c | 1.85 e | 14.59 d |
| Semirom | 0.07 de | 586.83 bc | 158.58 cde | 94.32 d | 23.59 c | 0.37 fg | 15.77 cd | 16.79 b | 0.09 j |
| Neishabour | 0.068 de | 611.81 bc | 170.10 bcd | 106.41 cd | 25.74 bc | 0.6 f | 16.5 c | 1.96 e | 15.69 c |
| Zarand | 0.064 e | 674.49 a | 146.10 ef | 99.42 cd | 19.46 d | 1.69 d | 11.63 f | 14.22 c | 9.15 h |
| Estahbanat | 0.079 c | 690.82 a | 182.19 b | 122.43 b | 29.81 a | 2.65 c | 14.65 d | 10 d | 15.07 cd |
| Anonymous | 0.074 cd | 620.68 b | 175.77 bc | 111.99 bc | 25.99 b | 1.16 e | 15.08 d | 1.54 e | 19.03 b |
| Birjand | 0.043 g | 709.24 a | 204.81 a | 142.05.35 ab | 24.09 bc | 0.07 h | 18.19 b | 0.0 f | 14.23 de |
| Kermanshah | 0.087 b | 479.45 d | 138.60 f | 93.6 d | 10.49 f | 0.0 h | 12.92 e | 0.0 f | 12.79 fg |
| Varamin | 0.103 a | 402.26 e | 151.17 def | 93.3 d | 8.23 f | 80.7 a | 6.62 g | 0.0 f | 60.09 a |
| Yazd | 0.070 de | 581.76 c | 174.00 bc | 107.01 cd | 21.22 d | 1.42 e | 13.06 e | 0.0 f | 12.47 g |
| Hamadan | 0.056 f | 686.06 a | 216.84 a | 142.46 a | 28.88 a | 0.13 gh | 21.19 a | 17.97 a | 5.9 i |

Phe: Phenol; Fla: Flavonoid; Chlo: Chlorogenic acid; Fer: Ferulic acid; Caf: Caffeic acid; Van: Vanillic acid; Sy: Syringic acid.

* and ** Significantly at the probability level of %5 and %1, respectively.

*Mean comparison was performed by LSD method at 5% probability. Columns with similar letters did not differ significantly.

Table S5 (continued).

| Phytochemical traits | | | | | | | | | |
| --- | --- | --- | --- | --- | --- | --- | --- | --- | --- |
| Accessions | *p*-cou | Her | Cou | Lut | Api | Chi | Gall | Nar | Que |
| Isfahan | 0.0 h | 78.36 b | 0.0 i | 109.73 e | 0.0 c | 0.0 d | 3.71 c | 0.0 c | 3.58 a |
| Abadeh | 0.0 h | 32.32 h | 21.37 c | 111.09 e | 0.0 c | 0.0 d | 4.41 b | 0.0 c | 0.0 c |
| Semirom | 6.62 e | 45.32 g | 8.22 d | 71.81 g | 1.53 a | 0.0 d | 0.0 d | 0.0 c | 0.0 c |
| Neishabour | 0.0 h | 28.82 i | 25.65 a | 239.18 c | 0.0 c | 0.0 d | 0.0 d | 4.07 a | 1.18 b |
| Zarand | 7.56 d | 33.62 h | 3.72 fg | 61.25 h | 0.55 b | 0.2 c | 3.76 c | 0.0 c | 0.0 c |
| Estahbanat | 10.81 b | 47.66 f | 3.32 g | 73.14 g | 0.0 c | 0.0 d | 0.0 d | 3.54 b | 0.0 c |
| Anonymous | 5.37 f | 52.22 e | 4.12 f | 85.66 f | 0.0 c | 0.0 d | 0.0 d | 0.0 c | 0.0 c |
| Birjand | 4.63 g | 55.04 d | 5.22 e | 69.07 g | 0.0 c | 4.23 a | 0.0 d | 0.0 c | 3.93 a |
| Kermanshah | 25.83 a | 78.25 b | 24.95 b | 465.18 b | 0.0 c | 2.58 b | 0.0 d | 0.0 c | 0.0 c |
| Varamin | 25.63 a | 107.54 a | 0.78 h | 591.49 a | 0.0 c | 0.16 cd | 0.0 d | 0.0 c | 0.0 c |
| Yazd | 8.59 c | 26.64 j | 3.39 g | 196.69 d | 0.0 c | 0.0 d | 0.0 d | 0.0 c | 0.0 c |
| Hamadan | 6.57 e | 62.61 c | 5.25 e | 107.36 e | 0.0 c | 0.0 d | 5.12 a | 0.0 c | 0.88 b |

*p*-cou: Para-coumaric acid; Her: Herniarin; Cou: Coumarin; Lut: Luteolin; Api: Apigenin; Chi: Chicoric acid; Gall: Gallic acid; Nar: Naringenin; Que: Quercetin

*Mean comparison was performed by LSD method at 5% probability. Columns with similar letters did not differ significantly.

Table S6. The mean comparison of interaction effects of different water deficit levels and tarragon accessions on phytochemical traits.

| Phytochemical traits | | | | | | | | | |
| --- | --- | --- | --- | --- | --- | --- | --- | --- | --- |
| 100% of Field capacity | | | | | | | | | |
| Accessions | DPPH (IC50) | FRAP | Phe | Fla | Chlo | Fer | Caf | Van | Syr |
| Isfahan | 0.078 f-i | 496.01 pq | 158.07 i-n | 95.17 i-m | 19.62 i-l | 19 d | 12.9 k-n | 10.88 g | 11.69 k |
| Abadeh | 0.109 a | 595.94 h-m | 106.92 o | 63.45 n | 37.2 ab | 0 r | 20.39 de | 4.094 j | 12.14 k |
| Semirom | 0.081 fg | 539.84 m-p | 159.48 i-n | 96.36 i-m | 34.32 bc | 0.523 pq | 16.28 ghi | 23.22 b | 0.288 o |
| Neishabour | 0.078 f-j | 587.24 i-n | 162.42 h-n | 105.69 g-m | 24.44 fgh | 0.668 op | 13.74 klm | 4.41 j | 11.42 k |
| Zarand | 0.072 g-l | 603.75 h-m | 131.93 no | 86.85 lmn | 27.76 def | 2.457 ij | 13.61 klm | 20.17 c | 7.185 lm |
| Estahbanat | 0.086 def | 702.98 a-e | 173.22 e-k | 111.48 e-l | 38.48 a | 2.031 jk | 8.94 pqr | 26.71 a | 5.146 n |
| Anonymous | 0.084 efg | 586.59 i-n | 169.95 f-k | 105.09 g-m | 22.7 ghi | 0.406 pqr | 10.03 o-r | 0.762 lm | 7.994 l |
| Birjand | 0.048 a-d | 679.55 a-g | 204.72 b-e | 133.11 a-e | 17.3 klm | 0 r | 11.81 mno | 0 m | 7.914 l |
| Kermanshah | 0.096 bcd | 502.34 opq | 136.02 l-o | 86.94 lmn | 12.8 nop | 0 r | 22.12 bcd | 0 m | 13.64 ij |
| Varamin | 0.111 a | 383.09 r | 140.82 k-n | 88.32 klm | 4.9 r | 104.2 a | 4 s | 0 m | 47.85 c |
| Yazd | 0.080 f-i | 562.06 l-o | 157.83 i-n | 87.96 lmn | 17.3 klm | 0.257 pqr | 8 r | 0 m | 11.8 k |
| Hamadan | 0.064 k-n | 659.61 b-h | 200.55 b-g | 130.77 a-f | 33.77 bc | 0 r | 25.39 a | 19.87 cd | 7.435 lm |
| 80% of Field capacity | | | | | | | | | |
| Isfahan | 0.069 h-l | 501.62 opq | 55.35 h-m | 106.83 f-m | 16.55 lmn | 17.81 e | 12.53 lmn | 8.793 i | 12.2 jk |
| Abadeh | 0.105 ab | 654.05 c-h | 157.53 i-n | 97.71 i-m | 23.02 ghi | 0.425 pqr | 14.78 ijk | 1.46 kl | 17.76 g |
| Semirom | 0.068 i-l | 580.02 j-n | 159.57 i-n | 82.65 mn | 21.49 hij | 0 r | 18.32 efg | 16.05 e | 0 o |
| Neishabour | 0.065 k-m | 603.06 h-m | 167.49 g-l | 100.47 h-m | 21.18 h-k | 1.132 mn | 14.02 jkl | 0 m | 16.18 h |
| Zarand | 0.063 k-m | 694.58 a-f | 129.93 k-n | 92.19 j-m | 18.94 i-m | 1.04 no | 11.01 nop | 14.4 f | 14.22 i |
| Estahbanat | 0.077 f-j | 716.54 abc | 190.32.44 c-i | 131.64 a-e | 27.13 ef | 1.94 kl | 16.05 hij | 1.746 k | 20.29 f |
| Anonymous | 0.073 g-k | 645.58 d-i | 193.11 c-h | 114.24 e-j | 30.76 cde | 3.001 h | 17.69 fgh | 2.04 k | 23.18 e |
| Birjand | 0.040 p | 722.21 ab | 206.85 a-d | 152.25 a | 30.57 cde | 0.219 pqr | 22.71 bc | 0 m | 17.66 g |
| Kermanshah | 0.085 def | 445.02 qr | 133.5 mno | 91.98 j-m | 9.54 pq | 0 r | 8.37 qr | 0 m | 10.89 k |
| Varamin | 0.102 abc | 400.39 r | 155.01 j-n | 93.09 j-m | 12.38 op | 56.76 c | 10.24 opq | 0 m | 72.86 a |
| Yazd | 0.069 h-l | 566.05 k-o | 169.2 g-k | 106.53 f-m | 25.86 fg | 1.515 lm | 17.7 fgh | 0 m | 12.k |
| Hamadan | 0.055 no | 690.98 a-f | 228.6 ab | 149.34 ab | 31.25 cd | 0 r | 24 ab | 19.18 d | 5.062 n |
| 60% of Field capacity | | | | | | | | | |
| Isfahan | 0.069 h-l | 530.8 nop | 180.75 d-j | 113.91 e-j | 11.57 op | 16.46 f | 8.91 pqr | 10 h | 16.88 gh |
| Abadeh | 0.093 cde | 603.55 h-m | 239.9 a | 150.21 ab | 18.25 j-m | 0 r | 14.57 i-l | 0 m | 13.87 i |
| Semirom | 0.062 k-n | 640.64 e-j | 156.66 j-n | 103.95 g-m | 14.98 mno | 0.595 op | 12.72 k-n | 11.12 g | 0 o |
| Neishabour | 0.061 lmn | 645.11 d-i | 180.42 d-j | 113.01 e-k | 31.62 cd | 0 r | 21.75 cd | 1.473 kl | 19.47 f |
| Zarand | 0.057 mno | 725.16 a | 164.43 h-n | 119.22 d-i | 11.69 op | 1.576 lm | 10.28 opq | 8.093 l | 6.065 mn |
| Estahbanat | 0.072 g-k | 652.93 c-h | 183.03 d-j | 124.2 c-h | 23.83 fgh | 3.98 g | 18.97 ef | 1.547 kl | 19.78 f |
| Anonymous | 0.067 j-m | 629.87 f-k | 164.25 h-n | 116.61 d-j | 24.52 fgh | 0.1 qr | 17.55 fgh | 1.823 k | 25.94 d |
| Birjand | 0.040 p | 725.96 a | 202.92 b-f | 139.71 a-d | 24.42 fgh | 0 r | 20.07 de | 0 m | 17.14 gh |
| Kermanshah | 0.080 fgh | 491 pq | 146.25 k-n | 101.88 g-m | 9.15 pq | 0 r | 8.27 qr | 0 m | 13.85 i |
| Varamin | 0.096 bcd | 423.29 r | 157.68 i-n | 98.52 i-m | 7.42 qr | 81.2 b | 5.63 s | 0 m | 59.57 b |
| Yazd | 0.064 k-m | 617.18 g-l | 195 c-h | 126.51 b-g | 20.51 h-l | 2.487 i | 13.48 klm | 0 m | 13.63 ij |
| Hamadan | 0.049 op | 707.53 a-d | 221.37 abc | 147.06 abc | 21.63 hij | 0.398 pqr | 14.21 i-l | 14.86 f | 5.208 n |

Phe: Phenol; Fla: Flavonoid; Chlo: Chlorogenic acid; Fer: Ferulic acid; Caf: Caffeic acid; Van: Vanillic acid; Sy: Syringic acid.

* and ** Significantly at the probability level of %5 and %1, respectively.

*Mean comparison was performed by LSD method at 5% probability. Columns with similar letters did not differ significantly.

Table S6 (continued).

| Phytochemical traits | | | | | | | | | |
| --- | --- | --- | --- | --- | --- | --- | --- | --- | --- |
| 100% of Field capacity | | | | | | | | | |
| Accessions | *p*-cou | Her | Cou | Lut | Api | Chi | Gall | Nar | Que |
| Isfahan | 0p | 63.22 gh | 0 t | 83.45 n-q | 0.0 c | 0.0 g | 3.59 cd | 0.0 f | 2.41 cd |
| Abadeh | 0p | 18.03 t | 34.98 c | 51.37 tuv | 0.0 c | 0.0 g | 4.5 b | 0.0 f | 0.0 f |
| Semirom | 8.677 g | 21.54 qrs | 19.04 e | 53.33 stu | 0.31 c | 0.0 g | 0.0 e | 0.0 f | 0.0 f |
| Neishabour | 0p | 21.54 qrs | 46.63 a | 214.8 g | 0.0 c | 0.0 g | 0.0 e | 7.9 a | 0.33 ef |
| Zarand | 8.436 gh | 18.23 st | 4.11 mn | 40.11 v | 0.23 c | 0.6 f | 3.99 bcd | 0.0 f | 0.0 f |
| Estahbanat | 19.1 d | 25.09 p | 0.815 rs | 47.6 uv | 0.0 c | 0.0 g | 0.0 e | 2.72 d | 0.0 f |
| Anonymous | 2.984 n | 40.12 mn | 5.53 k | 74.58 qr | 0.0 c | 0.0 g | 0.0 e | 0.0 f | 0.0 f |
| Birjand | 1.659 o | 37.6 n | 2.47 p | 51.02 tuv | 0.0 c | 3.33 c | 0.0 e | 0.0 f | 2.03 d |
| Kermanshah | 33.29 a | 34.15 o | 42.22 b | 197.3 h | 0.0 c | 2.26 e | 0.0 e | 0.0 f | 0.0 f |
| Varamin | 18.45 d | 94.41 c | 0.92 r | 517.4 d | 0.0 c | 0.0 g | 0.0 e | 0.0 f | 0.0 f |
| Yazd | 7.722 hi | 21.54 qrs | 3.65 no | 184.3 i | 0.0 c | 0.0 g | 0.0 e | 0.0 f | 0.0 f |
| Hamadan | 6.37 jkl | 17.82 t | 8.43 i | 60.98 st | 0.0 c | 0.0 g | 4.37 b | 0.0 f | 0.12 ef |
| 80% of Field capacity | | | | | | | | | |
| Isfahan | 0p | 84.27 d | 0 t | 110.6 m | 0.0 c | 0.0 g | 3.5 cd | 0.0 f | 2.8 c |
| Abadeh | 0p | 19.39 rst | 12.53 h | 121.9 lm | 0.0 c | 0.0 g | 4.27 b | 0.0 f | 0.0 f |
| Semirom | 5.714 klm | 44.19 l | 3.97 mn | 82.44 opq | 1.39 b | 0.0 g | 0.0 e | 0.0 f | 0.0 f |
| Neishabour | 0 p | 22.59 pqr | 13.29 g | 239.3 f | 0.0 c | 0.0 g | 0.0 e | 2.04 e | 0.76 e |
| Zarand | 8.12 gh | 32.21 o | 3.96 mn | 63.56 rs | 0.24 c | 0.0 g | 3.93 bcd | 0.0 f | 0.0 f |
| Estahbanat | 6.72 j | 55.29 j | 5 kl | 79.79 pq | 0.0 c | 0.0 g | 0.0 e | 4.42 b | 0.0 f |
| Anonymous | 7.69 hi | 39.51 mn | 4.44 lm | 87.79 nop | 0.0 c | 0.0 g | 0.0 e | 0.0 f | 0.0 f |
| Birjand | 6.694 j | 61.78 hi | 7.93 i | 62 st | 0.0 c | 5.38 a | 0.0 e | 0.0 f | 3.76 b |
| Kermanshah | 27.25 b | 96.41 c | 20.4 d | 539.7 c | 0.0 c | 2.54 e | 0.0 e | 0.0 f | 0.0 f |
| Varamin | 33.12 a | 103.6 b | 1.33 qr | 603.6 b | 0.0 c | 0.0 g | 0.0 e | 0.0 f | 0.0 f |
| Yazd | 10.02 f | 24.63 pq | 3.77 mno | 193.3 hi | 0.0 c | 0.0 g | 0.0 e | 0.0 f | 0.0 f |
| Hamadan | 6.527 jk | 85.42 d | 6.42 j | 129.4 kl | 0.0 c | 0.0 g | 5.17 a | 0.0 f | 0.12 ef |
| 60% of Field capacity | | | | | | | | | |
| Isfahan | 0 p | 87.59 d | 0 t | 135.1 k | 0.0 c | 0.0 g | 4.04 bc | 0.0 f | 5.52 a |
| Abadeh | 0 p | 59.55 i | 16.62 f | 160 j | 0.0 c | 0.0 g | 4.46 b | 0.0 f | 0.0 f |
| Semirom | 5.487 lm | 70.26 f | 1.67 q | 79.66 pq | 2.89 a | 0.0 g | 0.0 e | 0.0 f | 0.0 f |
| Neishabour | 0 p | 42.35 lm | 17.04 f | 263.5 e | 0.0 c | 0.0 g | 0.0 e | 2.26 e | 2.45 cd |
| Zarand | 6.145 j-m | 49.44 k | 3.11 op | 80.1 pq | 1.19 b | 0.0 g | 3.34 d | 0.0 f | 0.0 f |
| Estahbanat | 6.624 j | 62.63 ghi | 4.15 mn | 92.04 no | 0.0 c | 0.0 g | 0.0 e | 3.48 c | 6.01 a |
| Anonymous | 5.462 m | 77.04 e | 2.41 p | 96.63 n | 0.0 c | 0.0 g | 0.0 e | 0.0 f | 0.0 f |
| Birjand | 5.547 lm | 65.76 g | 5.27 k | 94.2 n | 0.0 c | 3.98 b | 0.0 e | 0.0 f | 0.0 f |
| Kermanshah | 16.98 e | 104.2 b | 12.24 h | 658.6 a | 0.0 c | 2.93 d | 0.0 e | 0.0 f | 0.0 f |
| Varamin | 25.35 c | 124.6 a | 0.11 st | 653.5 a | 0.0 c | 0.49 f | 0.0 e | 0.0 f | 0.0 f |
| Yazd | 8.043 gh | 33.75 o | 2.75 p | 212.6 g | 0.0 c | 0.0 g | 0.0 e | 0.0 f | 0.0 f |
| Hamadan | 6.836 ij | 84.61 d | 0.93 r | 131.7 kl | 0.0 c | 0.0 g | 5.82 a | 0.0 f | 0.0 f |

*p*-cou: Para-coumaric acid; Her: Herniarin; Cou: Coumarin; Lut: Luteolin; Api: Apigenin; Chi: Chicoric acid; Gall: Gallic acid; Nar: Naringenin; Que: Quercetin

*Mean comparison was performed by LSD method at 5% probability. Columns with similar letters did not differ significantly.

Table S7. Pearson correlation coefficient between phytochemical traits on tarragon accessions.

|  | Phe | Fla | FRAP | IC_50_ | Chlo | Syr | Fer | Van | Caf | *p*-cou | Her | Cou | Lut |
| --- | --- | --- | --- | --- | --- | --- | --- | --- | --- | --- | --- | --- | --- |
| Phe | 1 |  |  |  |  |  |  |  |  |  |  |  |  |
| Fla | 0.953** | 1 |  |  |  |  |  |  |  |  |  |  |  |
| FRAP | 0.645* | 0.709** | 1 |  |  |  |  |  |  |  |  |  |  |
| IC50 | -0.607* | -0.673* | -0.667* | 1 |  |  |  |  |  |  |  |  |  |
| Chlo | 0.685* | 0.62* | 0.868** | -0.435 | 1 |  |  |  |  |  |  |  |  |
| Syr | -0.251 | -0.259 | -0.624* | 0.562 | -0.556 | 1 |  |  |  |  |  |  |  |
| Fer | -0.29 | -0.33 | -0.71** | 0.519* | -0.656* | 0.929** | 1 |  |  |  |  |  |  |
| Van | 0.187 | 0.163 | 0.365 | -0.342 | 0.334 | -0.505 | -0.233 | 1 |  |  |  |  |  |
| Caf | 0.749** | 0.719** | 0.752** | -0.567 | 0.807** | -0.672* | -0.726** | 0.285 | 1 |  |  |  |  |
| *p*-cou | -0.46 | -0.346 | -0.603* | 0.438 | -0.699* | 0.551 | 0.549 | -0.255 | -0.554 | 1 |  |  |  |
| Her | -0.155 | -0.113 | -0.681* | 0.321 | -0.694* | 0.646* | 0.749** | -0.104 | -0.485 | 0.652* | 1 |  |  |
| Cou | -0.274 | -0.257 | 0.072 | 0.259 | 0.023 | 0.204 | -0.347 | -0.327 | 0.273 | -0.005 | -0.271 | 1 |  |
| Lut | -0.499 | -0.478 | -0.831** | 0.586* | -0.791** | 0.739** | 0.709** | -0.509* | -0.606* | 0.816** | 0.655* | 0.241 | 1 |

Phe: Phenol; Fla: Flavonoid; IC50: DDPH; Chlo: Chlorogenic acid; Sy: Syringic acid; Fer: Ferulic acid; Van: Vanillic acid; Caf: Caffeic acid;

*p*-cou: *p*-coumaric acid; Her: Herniarin; Cou: Coumarin;

* and ** Significantly at the probability level of %5 and %1, respectively.

Table S8. Specifications related to the origin of tarragon samples collected in this study

| Accessions | Province | City | Latitude & Longitude | Above sea level (m) |
| --- | --- | --- | --- | --- |
| Isfahan | Isfahan | Isfahan | 32° 38′ 41″ N, 51° 40′ 3″ E | 1574 |
| Abadeh | Fars | Abadeh | 31° 9′ 39″ N, 52° 39′ 2″ E | 1890 |
| Semirom | Isfahan | Semirom | 31° 24′ 51″ N, 51° 34′ 10″ E | 2000 |
| Neishabour | Razavi Khorasan | Neishabour | 36° 12′ 48″ N, 58° 47′ 45″ E | 1250 |
| Zarand | Kerman | Zarand | 30° 48′ 46″ N, 56° 33′ 50″ E | 1656 |
| Estahbanat | Fars | Estahbanat | 29° 7′ 36″ N, 54° 2′ 32″ E | 1767 |
| Anonymous | - | - | - | - |
| Birjand | South Khorasan | Birjand | 32° 52′ 0″ N, 59° 12′ 0″ E | 1491 |
| Kermanshah | Kermanshah | Kermanshah | 34° 18′ 51″ N, 47° 3′ 54″ E | 1350 |
| Varamin | Tehran | Varamin | 35° 19′ 27″ N, 51° 38′ 45″ E | 920 |
| Yazd | Yazd | Yazd | 31° 53′ 50″ N, 54° 22′ 4″ E | 1216 |
| Hamadan | Hamadan | Hamadan | 34° 47′ 54″ N, 48° 30′ 54″ E | 1850 |

Table S9. The measured values of the selected soil properties

| No | Soil properties | Unit | No | Soil properties | Unit |
| --- | --- | --- | --- | --- | --- |
| 1 | Total nitrogen (%) | 0.09 | 7 | Sand (%) | 22.6 |
| 2 | Exchangeable Potassium (ppm) | 696.0 | 8 | Clay (%) | 46.2 |
| 3 | Available Phosphorus (ppm) | 104.0 | 9 | Silt (%) | 31.2 |
| 4 | Organic matter (gr/kg) | 6.25 | 10 | Calcium carbonate (%) | 5.9 |
| 5 | Acidity (pH) | 6.8 | 11 | Calcium (ppm) | 400 |
| 6 | Electrical conductivity (ds/m) | 1.26 | 12 | Organic carbon (%) | 0.93 |

Table S10. Climatic conditions of tarragon cultivation area.

| Parameters | Average minimum temperature  (◦C) | Average maximum temperature  (◦C) | Average monthly temperature  (◦C) | | | Average monthly rainfall  (mm) | | Average percentage of relative humidity | Average minimum temperature  (◦C) | Average maximum temperature  (◦C) | | | Average monthly temperature  (◦C) | | Average monthly rainfall  (mm) | Average percentage of relative humidity |
| --- | --- | --- | --- | --- | --- | --- | --- | --- | --- | --- | --- | --- | --- | --- | --- | --- |
|  |  | | | | | | | | | | | | | | | |
|  | April | | | | | | | | May | | | | | | |  |
|  | 9.06 | 22.27 | 16.01 | | | 0.69 | | 49.77 | 14.59 | 28.85 | | | 21.68 | | 0.41 | 36.94 |
|  |  | | | | | | | | | | | | | | | |
|  | June | | | | | | | | July | | | | | | | |
|  | 18.37 | 34.9 | | 26.96 | 0.17 | | 29.9 | | 21 | 36.9 | 28.80 | | | | 0.25 | 30.33 |
|  |  | | | | | | | | | | | | | | | |
|  | August | | | | | | | | September | | | | | | | |
|  | 20.33 | 35.17 | 27.68 | | | 0 | | 28.56 | 16.50 | 31.00 | | 23.82 | | 0.09 | | 33.45 |
